# Co-cultivation dynamics of the filamentous microorganisms *Aspergillus niger* and *Streptomyces coelicolor* in shake flasks

**DOI:** 10.1186/s12934-026-03015-9

**Published:** 2026-05-06

**Authors:** Tolue Kheirkhah, Fangxing Zhang, Fabia Jaeger, Alexander Gantenbein, Peter Neubauer, Heiko Briesen, Stefan Junne

**Affiliations:** 1 Institute of Biotechnology, Chair of Bioprocess Engineering, Technische Universität Berlin, Ackerstraße 76 ACK24, 13355 Berlin, Germany; 2 School of Life Sciences Weihenstephan, Chair of Process Systems Engineering, Technische Universität München, Gregor-Mendel-Straße 4, 85354 Freising, Germany; 3 Department of Chemistry and Bioscience, Aalborg University, Niels Bohrs Vej 8, 6700 Esbjerg, Denmark

**Keywords:** Filamentous microorganisms, Co-culture, Automated image analysis, Population dynamics, Shake flask cultivation, Shear force

## Abstract

**Background:**

The co-cultivation of filamentous fungi and actinobacteria is challenging due to their complex growth interactions. This study investigates how key parameters, such as inoculation strategy, glutamic acid concentration, hydrodynamic stress, and dissolved oxygen, influence the growth dynamics between *Aspergillus niger* and *Streptomyces coelicolor* in shake flask co-cultures. Recognizing the crucial role of macromorphology in filamentous microorganisms, an automated image analysis pipeline was developed to quantitatively assess the heterogeneity and reproducibility of each population.

**Results:**

Simultaneous growth was achieved when both microorganisms were inoculated in pelleted form, whereas spore inoculation led to complete *A. niger* dominance. At 1:2 and higher inoculation ratios (fungus to bacteria), *S. coelicolor* could compete effectively. While *A. niger* growth-maintained dominance at 136 and 250 rpm (1:1), *S. coelicolor* growth outcompeted the fungus at 60 rpm, a shift attributed to a reduced oxygen transfer rate. Notably, only the highest shear forces (250 rpm) produced homogeneous, reproducible fungal pellet populations. Overall, bottom-baffled flasks enhanced reproducibility compared to non-baffled flasks.

**Conclusion:**

It is possible to regulate the growth of *S. coelicolor* and *A. niger* in a co-culture by the aforementioned parameters. Among these, the inoculation ratio is most important to achieve different dynamics. A quantitative analysis of morphology development while optimising inoculation strategies provides a foundation for designing co-culture experiments that achieve balanced and reproducible growth.

**Supplementary Information:**

The online version contains supplementary material available at 10.1186/s12934-026-03015-9.

## Background

Filamentous microorganisms, such as fungi of the genus *Aspergillus* and bacteria of the genus *Streptomyces*, are well known for their high production capacity and ability to secrete enzymes and secondary metabolites [1, 2]. Owing to their rich repertoire of biosynthetic gene clusters, co-cultivation of organisms from these two groups presents a promising strategy for expanding the diversity of detectable metabolites [3, 4]. This occurs through interspecies interactions, which can trigger or inhibit metabolic pathways and activate silent genes, potentially leading to the accumulation of rare secondary compounds or enzymes, which are often undetectable in axenic cultures [5, 6]. Co-cultivation may involve cooperative interactions, where chemical signaling and physical contacts enable the exchange of nutrients and cofactors [7]. For example, co-cultivation of the two filamentous fungi *Aspergillus niger* and *Trichoderma reesei* has been shown to enhance cellulase activity compared to *A. niger* axenic cultures synergistically [8]. Conversely, competitive interactions can happen in co-cultures, where strains compete for limited resources or engage in direct antagonism. A notable case is the interaction between fungi and actinomycetes, which is primarily characterized by complex microbial offense and defense strategies, including the secretion of antimicrobial compounds and adaptive resistance mechanisms [4, 9].

Most co-culture studies have focused on biosynthetic pathways and metabolite production [6, 10]. However, achieving certain scenarios in filamentous co-cultivation systems requires careful consideration of morphology as each organism can adopt distinct structural forms, ranging from dispersed mycelia to compact pellets. It has been observed that the morphology of *A. niger* and *Streptomyces coelicolor* is closely linked to the accumulation of secondary metabolites. This was exemplified by enhanced enniatin B and actinorhodin production with compact pellets in the respective cultures [11, 12]. While it is possible to achieve a rather defined morphology under controlled cultivation conditions in axenic cultures [13, 14], maintaining such control in co-cultures of filamentous microorganisms is considerably more difficult [15]. Such systems often result in heterogeneous morphologies and limited reproducibility, posing a major challenge for process consistency and scale-up. For example, *Aspergillus terreus* morphology shifted from predominantly pellet formation to heterogeneous clumps when co-cultivated with *Streptomyces rimosus* [16]. Such changes may significantly influence population dynamics by affecting growth kinetics, metabolic production profiles, and the overall balance of the two populations.

Although morphology is a crucial factor, qualitative microscopic observations have primarily been relied on in most studies [16]. Incorporating quantitative data, however, has proven effective for tracking dynamic processes such as pellet agglomeration and breakage through size distribution analysis, as well as assessing the morphological structure itself [17, 18]. In co-cultivation studies, the growth of the cultures is often monitored through measurements such as cell dry weight and flow cytometry, without differentiating between the individual species unless they are specifically labeled [19, 20]. Frequently, the dominance of one species is inferred from the concentrations of specific metabolites, yet this indirect approach provides limited insight into population dynamics [6, 21, 22].

To overcome these limitations, the implementation of an automated image analysis pipeline, capable of classifying microorganisms based on features such as color patterns, morphology, and structural details enable a quantitative assessment of each species’ growth. This is particularly relevant in the context of pellet formation, as pellets often represent the preferred morphology for the production of secondary metabolites with filamentous microorganisms [23, 24]. Quantitative morphological analysis not only enables evaluation of reproducibility and morphological heterogeneity but also facilitates a deeper understanding of how environmental factors influence co-culture performance.

Factors such as inoculum volume, shear stress, pH, dissolved oxygen concentration, and medium composition can directly affect microbial morphology and, consequently, product formation. They can therefore be adjusted to steer cultivation conditions [13, 25–27]. It is important to note that in co-cultures, these environmental factors may affect each strain differently [10], potentially leading to alterations in population dynamics. This effect is particularly relevant in filamentous cultures due to their morphological sensitivity.

Given that each strain has distinct requirements to achieve certain growth characteristics, co-culture inoculation strategies should be tailored to prevent the rapid outgrowth of one partner [10]. Depending on the specific objectives of the co-cultivation, such as maximizing metabolite production, promoting synergistic interactions, or preserving a balance of the species, different inoculation strategies may be employed. Successful scenarios include varying inoculation ratios [28, 29], inoculating the organisms at different growth stages [10, 30], modifying the composition of the cultivation medium [29], and applying a time-shifted inoculation to give a slower-growing organism a competitive advantage over its faster-proliferating partner [5]. For example, inoculating *S. coelicolor* after 72 h of axenic growth of *A. niger* enhanced bioactive compound production in *S. coelicolor.* Nevertheless, this strategy also led to the degradation of fungal pellets, likely triggered by an antagonistic activity from the bacterial strain [3].

The present study investigates the dynamics between the filamentous fungus *A. niger* and the actinobacterium *S. coelicolor* under various cultivation conditions in shake flask co-cultures. Specifically, the influence of the inoculation ratio, gas mass transfer, and the shear force regime, modulated through various shaking frequencies and filling volumes, on the co-culture performance and reproducibility is examined. To obtain quantitative data, an automated image analysis pipeline that assesses microbial morphology and growth patterns is described. The overarching goal is to achieve a robust workflow that enables balanced growth and homogeneous morphology with high reproducibility.

## Methods

### Strain and inoculum preparation

The experiments were conducted with the strains *Aspergillus niger* ÖV4.10 [31] and *Streptomyces coelicolor* M145 (DSMZ - German Collection of Microorganisms and Cell Cultures GmbH, Braunschweig, Germany). Both strains were preserved as cryostocks at -80 °C in 50% (v v^− 1^) glycerol in deionized water.

The salt base of the “SSBM-P” medium [32], which was designed to promote the growth of *S. coelicolor*, was used for both axenic and co-culture experiments. D-glucose (10 g L⁻¹) and L-glutamic acid (10 g L⁻¹) were added as carbon and nitrogen sources. These components ensure sufficient biomass to analyze secondary metabolite production when triggered by phosphate depletion (initial C/P ratio of 200:1 mol mol^− 1^). Only in the initial experiment (for choosing the inoculum type), 1.5 g L^− 1^ glutamic acid was used. All solutions were sterilized by autoclaving at 121 °C for 20 min. The pH of the medium was adjusted to 6.0 or 7.0, as appropriate for the experiment, using 1 M HCl or 1 M NaOH before sterilization.

*A. niger* agar plates were prepared using complete medium (CM) agar, as described in Kheirkhah et al. [13]. For *S. coelicolor*, soy flour mannitol (SFM) agar was used, consisting of 20 g L^− 1^ of each soy flour, mannitol, and agar. Petri dishes were incubated at 30 °C for 10 days. Spores were harvested by rinsing the plates with sterile 0.9% (w v^− 1^) NaCl, then filtering through Miracloth filters (Merck KGaA, Darmstadt, Germany) with a pore size of 25 μm to remove agar pieces before centrifugation at 8,000 × g for 10 min. *A. niger* spores were counted using a Thoma chamber. Since *S. coelicolor* spores were too small to count with a typical light microscope, they were plated on LB agar (containing per liter: 5 g casein peptone, 5 g yeast extract, 10 g NaCl, and 15 g agar) and quantified based on colony formation. A fresh *A. niger* spore suspension was prepared before each cultivation, while *S. coelicolor* pre-culture flasks were inoculated from a cryostock. CM was also used as a pre-culture medium for *A. niger*, and double-strength yeast tryptone medium (2× YT) for *S. coelicolor*, containing 16 g L^− 1^ tryptone, 10 g L^− 1^ yeast extract, and 5 g L^− 1^ NaCl. All ingredients were purchased from Carl Roth (Karlsruhe, Germany) and Sigma-Aldrich (Schnelldorf, Germany).

### Co-cultivation

Each co-culture experiment started with a pre-culture phase where 6.25 × 10⁵ spores mL^− 1^ of each partner were inoculated separately in 500 mL non-baffled Erlenmeyer flasks (NF): *A. niger* was grown for 12 h in CM, and *S. coelicolor* for 20 h in 2× YT until the end of the exponential growth phase. The filling volume V_L_ was 50 mL unless stated otherwise, and the shaking frequency was always set to 250 rpm. The medium was separated from cell pellets by sedimentation, after which the cells were rinsed, resuspended in fresh media (“SSBM-P”), and transferred to 500 mL Erlenmeyer flasks with or without baffles. The filling volume V_L_ of main cultures was always 100 mL, and the shaking frequency was set to 250 rpm except when this parameter was changed (Table 1). Each co-culture was inoculated using pellets from one pre-culture flask of each strain. To investigate the influence of different inoculation ratios on co-cultivation, six co-culture experiments were conducted with varying *A. niger* to *S. coelicolor* spore ratios: 1:1, 1:2, 1:5, 1:10, 1:100, and 1:1,000. The spore concentration was changed in the pre-culture (adjusting *A. niger* spore concentration while keeping the *S. coelicolor* spore concentration constant at 6.25 × 10⁵ spores mL^− 1^). A separate set of experiments was conducted in which the volume of the pre-culture was adjusted to 50, 75, or 100 mL, while maintaining the same initial number of spores in the subsequent culture as at a spore ratio of 1:1. To test the influence of varying initial concentrations of glutamic acid on the pellet size, 100 mL of “SSBM-P” medium with glutamic acid (0.5, 2.5, 5, and 10 g L^− 1^) was inoculated with 6.25 × 10⁵ *A. niger* spores mL^− 1^ in 500 mL NF. All these flask cultures were incubated at 30 °C and 250 rpm on an orbital shaker (incubator) with an amplitude of 50 mm. Co-culture experiments were performed in biological duplicates using both 500 mL Erlenmeyer glass flasks without baffles (NF) and polycarbonate sensor flasks with baffles (BF) (SFS-HP5, PreSens Precision Sensing GmbH, Regensburg, Germany). The baffled flasks had a bottom diameter of 100 mm, a total height of 180 mm, and a neck opening diameter of 40 mm. The flasks were equipped with four integrated baffles at the bottom, each 30 mm in length, and with built-in fluorescence-based pH and DO sensors for *online* monitoring. Therefore, the DO profiles were recorded non-invasively using the PreSens SFR Shake Flask Reader with pre-calibrated sensors, according to the manufacturer’s protocol. The NFs had the same total height and neck diameter as the BFs, but a button diameter of 108 mm. The following assumption for NF was applied to estimate the change of the oxygen transfer rates (OTRs) at different filling volumes among experiments [33]:

1 $$ \begin{aligned} {\mathrm{OTR}}_{{{\mathrm{max}}}} = & {\text{ 3}}.{\text{72 }} \times {\text{ 1}}0^{{ - {\mathrm{7}}}} \times {\text{ Osmol}}^{{0.0{\mathrm{5}}}} \times {\text{ n}}^{{({\mathrm{1}}.{\mathrm{18}} - {\mathrm{Osmol}}/{\mathrm{1}}0.{\mathrm{1}})}} \\ & \times {\text{ V}}_{{\mathrm{L}}} ^{{ - 0.{\mathrm{74}}}} \times {\text{ d}}_{0} ^{{0.{\mathrm{33}}}} \times {\text{ d}}^{{{\mathrm{1}}.{\mathrm{88}}}} \times {\text{ p}}_{{\mathrm{R}}} \times {\text{ y}}_{{{\mathrm{O2}}}} \\ \end{aligned} $$

The exponent of the shaking frequency n was assumed to be that of LB media (1.13) [33]. The pressure was p_R_ = 1.013 bar, the molar fraction of O_2_ in the gas phase y_O2_ = 0.2093.

In case of BFs, the following assumption was used, which was originally published about the same flask geometry [34]:

2 $${\mathrm{OT}}{{\mathrm{R}}_{{\mathrm{max}}}}={c^*} \times a~ \times {e^{ - 0.5~\left( {{{\left( {\frac{{n - ~{x_0}}}{b}} \right)}^2}+{{\left( {\frac{{{V_{max}} - {y_0}}}{c}} \right)}^2}} \right)}}$$

At a filling volume of 100 mL (20% (v v^− 1^)), x_0_ = 235.32, y_0_ = 276.87, a = 333.95, b = 59.23, and c = 347.81 [34]. The maximum solubility of oxygen in media at 30 °C is estimated to c* = 0.21 mmol L^− 1^. Empirical equations are described for Newtonian, low-viscosity fluids. Since the co- cultures in this study formed mainly pellets, any deviation from these assumptions is expected to have minimal impact on the relative comparison. The OTR for NF (500 mL total volume, 100 mL filling volume, 250 rpm) is 15 mmol (L h)^−1^ according to Eq. 1, while the OTR of cultivations in BF (500 mL total volume, 100 mL filling volume) is 55 mmol (L h)^−1^ (250 rpm), 14 mmol (L h)^−1^ (136 rpm) and 0.71 mmol (L h)^−1^ (60 rpm). By reducing the shaking frequency from 250 rpm to 136 rpm, and then to 60 rpm, the OTR was reduced to about 25% and 1.2% of the OTR at 250 rpm, respectively. OTR estimations were used to discuss results qualitatively.

### Analysis

All experiments were performed in biological duplicates. At each time point, two 5 mL samples were taken from each shake flask using cut plastic tips, providing a wider diameter for pellets. These two technical replicates were filtered on pre-dried filter paper using a vacuum pump and subsequently dried in an oven (50 °C) to determine the cell dry weight as described previously [13]. The corresponding filtrates were used to quantify glucose, kojic acid, and glutamic acid concentrations. Glucose was measured in an HPLC-RID (1200 series, Agilent Technologies GmbH, Waldbronn, Germany) with a HyperREZ XP Carbohydrate H^+^ column (Thermo Fisher Scientific Inc., Waltham, MA, USA) [13]. Kojic acid was quantified in an HPLC-DAD (1200 series, Agilent Technologies) with a Kinetex 2.6 μm phenyl-hexyl column (Phenomenex GmbH, Aschaffenburg, Germany) and 2% (v v^− 1^) acetonitrile containing 1% (v v^− 1^) acetic acid as eluent at a flow rate of 0.6 mL min^− 1^. The detection wavelength was set to 268 nm at 30 °C. The glutamic acid concentration was quantified with a Cedex Bio-HT^®^ analyzer (I&L-Biosystems GmbH, Koenigswinter, Germany) using the assay kit Glutamate V2 Bio HT. Error bars represent the standard deviation of biological replicates (*n* = 2) in all figures.

### Image acquisition

1 mL of suspension was collected at each sampling point and stored at -20 °C until further analysis. Differential interference contrast (DIC) microscopy (Leica Microsystems DM 5000 microscope, with a DFC365 FX CCD microscopy camera, Leica Microsystems GmbH, Wetzlar, Germany) was used for the qualitative analysis of the pellet morphology. For pellet diameter distribution, stereo microscopy with a Leica S8APO microscope was applied (Leica Microsystems GmbH, Wetzlar, Germany). Samples for imaging were prepared by the addition of 1 mL of the cell suspension sample to a Petri dish containing 9 mL of 0.9% NaCl (w/v) and 2 µL of 50% (v v^− 1^) Tween® 20 to reduce the surface tension. Images were captured at a total magnification of 12.5 × for DIC microscopy, while the stereo microscope was used at various magnifications, as indicated on the respective images.

### Automated image analysis and data processing

**Fig. 1:**
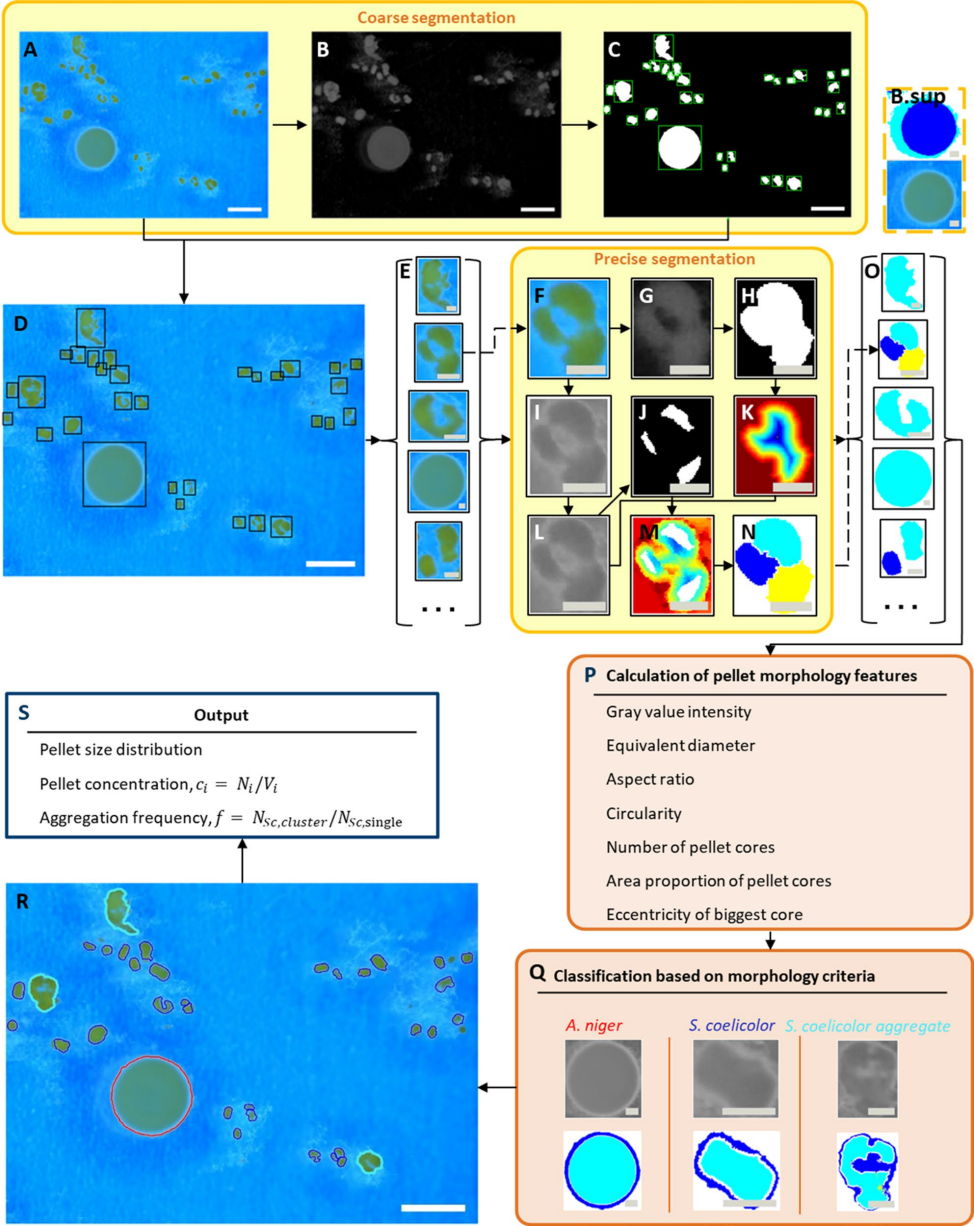
The automated image processing pipeline for detecting *Aspergillus niger* and *Streptomyces coelicolor* pellets. **A** Raw image acquired from the stereo microscope. **B** and **C** Coarse segmentation steps of objects in the image. **D**, **E** Extraction of coarsely segmented objects along their bounding boxes. **F**–**N** Precise segmentation steps for separating closely adjacent pellets. **O**–**P** Calculation of morphological feature parameters for classification. **Q** and **R** Classification of pellets and their result, where single *A. niger* pellets are marked in red, *S. coelicolor* single pellets in dark blue, and *S. coelicolor* aggregates in cyan. **S** Population-level information extracted from the image analysis pipeline. The white scale bar represents 1 mm, and the gray scale bar 0.2 mm

8-bit RGB image data (Fig. 1A) acquired from a stereo microscope were analyzed using MATLAB 2022a (MathWorks Inc., Natick, MA, USA) to segment and classify individual microorganisms. The segmentation of single pellets began with a coarse segmentation of objects. Since the hyphae around the pellets appeared bright against a blue background, the red channel of the image (Fig. 1B) was selected. The image was divided into three layers with thresholds obtained from the MATLAB function “multithresh” (Fig. 1B.sup): background (white), one-sided bright shadows around pellets caused by lateral lighting (light blue), and pellets (dark blue). A binary mask was created from the third layer and processed with filling steps (MATLAB functions “imopen”, “imclose”, “imfill”) to obtain a binarized image as shown in Fig. 1C. Objects larger than 0.0165 mm² were categorized as “potential pellets”. These objects were extracted along their bounding boxes (Fig. 1D) and saved as individual images for further processing (Fig. 1E).

From the extracted single images (Fig. 1F), precise pellet contours were obtained through a second multi-threshold binarization (MATLAB function “multithresh”) of the red channel (Fig. 1G), where the first level represented the background, the second level the shadow, and the third level the pellets. Since only the pre-segmented regions of the image are considered rather than the entire image, the threshold values differ from those used in the initial coarse segmentation of the objects described above. This resulted in a refined binary mask delineating the exact pellet contours (Fig. 1H). Next, a distance transformation (MATLAB function “bwdist”) was applied to the inverted pellet contour mask, calculating the distance of each pellet pixel to the outer edge. This resulting distance matrix was normalized between 0 and 1, with the highest values at the pellet border and the lowest at the morphological center (Fig. 1K).

In parallel, the extracted image (Fig. 1F) was converted into a gray value image by the MATLAB function “rgb2gray” (Fig. 1I), followed by a correction for location-dependent brightness variations using the CIDRE method (Corrected Intensity Distributions for Image Restoration and Enhancement), as described by Smith et al. [35]. Subsequently, a disk median filter with a radius equal to one-twentieth of the pellet diameter was applied to reduce noise in the grayscale image. The gray values in the final image were normalized between 0 and 1 (Fig. 1L).

After grayscale image correction and smoothing (Fig. 1L), the Otsu’s threshold binarization method [36] (MATLAB function “imbinarize”) was applied, allowing separation of the pellet core region, which is characterized by lower gray value intensities in the grayscale image (Fig. 1J).

As cultivation progressed, *S. coelicolor* and *A. niger* pellets exhibited significant size differences. To support the separation of closely adjacent pellets, the normalized values of the grayscale image (Fig. 1L) were added to the normalized distance matrix (Fig. 1K), and the pellet core regions (Fig. 1J) were imposed as the local minimum on that. The resulting image is shown as Fig. 1M. A marker-controlled watershed algorithm (MATLAB function “watershed”) was then applied to Fig. 1M to detect the boundary between closely adjacent pellets (Fig. 1N). Under typical shake flask cultivation conditions, *A. niger* usually forms large spherical pellets with a visible dark single pellet core surrounded by white hyphae. *S. coelicolor* grows to a smaller size, around 250 μm (diameter) instead, and may form agglomerates with an irregular shape and multiple cores. The dark pellet core represents a region with high hyphal density, as shown in the previous 3D structural analysis [37]. Microscopic images revealed that fungal pellets typically exhibited a smaller proportion of the core area and a higher average gray intensity across the whole pellet compared to pellets of *S. coelicolor*. Following precise pellet segmentation (Fig. 1O), the morphological feature parameters listed in Fig. 1P were calculated for each pellet. The corresponding formulas for the feature parameters are provided in Table S1 (appendix). These morphological characteristics were used to classify particles either as single *A. niger* pellets, single *S. coelicolor* pellets, or *S. coelicolor* aggregates (Fig. 1Q), based on the classification criteria shown in Fig. S1 in the appendix. The classification results are visualized with distinct colors for each class in the microscopic image (Fig. 1R). After completing the entire process, additional population-level parameters, such as pellet-size distribution, pellet concentration, and aggregation frequency, can be calculated for each species (Fig. 1S).

## Results

### Automated image analysis

An important part of this work was to develop an automated image analysis workflow to quantify morphological characteristics of the two protagonists individually from cell suspension samples of co-cultures. The assessment of morphology provides an overview of individual growth performance and supports the identification of suitable cultivation parameters. By identifying the bright edge in the grayscale image and using the morphological information from the distance matrix, even relatively small pellets attached to large ones were detected and differentiated without fragmenting large pellets. Correlation and principal component analysis (PCA) were performed on the morphological features. The correlation matrix (Table S2) shows that most features are weakly correlated (|r| < 0.4). The PCA results (Fig. S2) further demonstrate that the selected features quantify distinct aspects of pellet morphology. Based on the loading coefficients (Table S3), no principal component is dominated by a single feature, indicating that the feature is moderately correlated but not redundant, and captures complementary morphological information. A comparison of classification results for 45,819 pellets from 4,804 images acquired at different cultivation times, using manually annotated data, achieved an overall accuracy of 95% (Table S4).

### Inoculation workflow

**Fig. 2:**
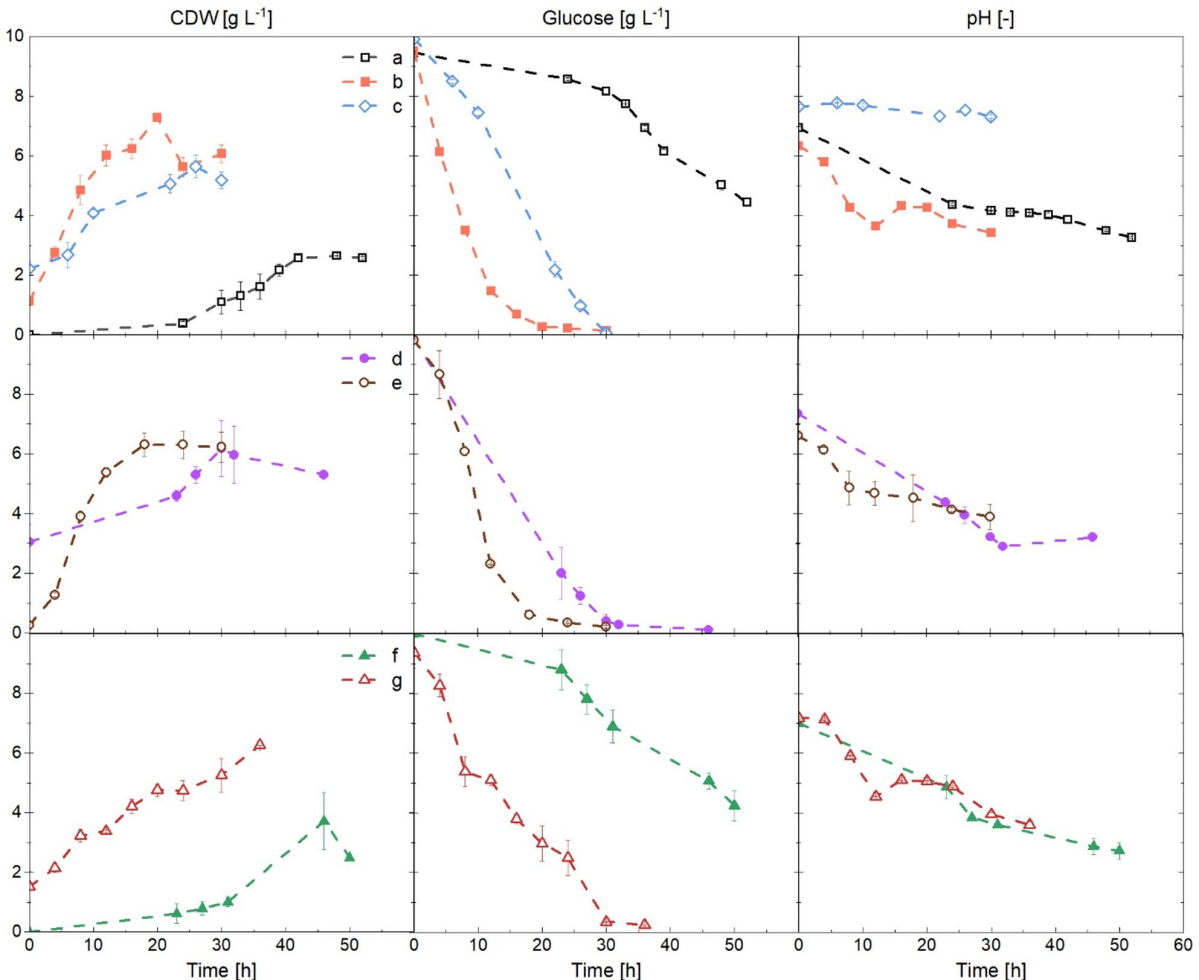
Comparison of cell dry weight (CDW), glucose concentration, and pH in axenic and co-cultures with various inoculation scenarios. **a** Axenic culture of *Aspergillus niger* starting from spores, **b** axenic culture of *A. niger* from pellets (pre-culture), **c** axenic culture of *Streptomyces coelicolor* from pellets (pre-culture), **d** co-culture of *A. niger* spores and *S. coelicolor* pellets (The initial CDW concentration for *S. coelicolor* pellets appears to be overestimated, likely due to inaccuracies during initial sampling), **e** co-culture of *A. niger* pellets and *S. coelicolor* spores that germinated, **f** co-culture of *A. niger* spores and *S. coelicolor* spores, **g** co-culture of *A. niger* pellets and *S. coelicolor* pellets

To establish an inoculation workflow, *A. niger* and *S. coelicolor* were cultivated using different inoculation scenarios. The microorganisms were inoculated into the co-culture either as spore suspensions or as pre-grown pellets from a pre-culture. Therefore, the initial objective was to identify which morphological state of the inoculum allows both organisms to grow simultaneously, without one organism dominating the culture from the outset. Growth dynamics and morphological interactions between the strains in co-culture were then compared with their respective axenic cultures. Figure 2 illustrates the growth curve, glucose consumption, and pH variations across the different cultivation conditions. The growth characteristics of all cultures, including specific growth rate (µ), substrate consumption rate (r_S_), specific consumption rate (q_S_), and biomass yield (Y_x/s_), are summarized in Table 1 and Fig. S4.

When inoculated with spores, *A. niger* had a low specific growth rate (µ = 0.10 h⁻¹) and a prolonged lag phase (Fig. 2a), while pellet inoculation, as expected, led to faster growth (µ = 0.30 h⁻¹) (Fig. 2b. Both cultures developed in pellet form, consistent with previous findings that *A. niger* forms pellets under a low shear force environment, typical also for non-baffled flasks [18, 38]. Y_x/s_ was rather similar between the two conditions (0.67 g biomass g⁻¹ glucose for pellet inoculation vs. 0.62 g biomass g⁻¹ glucose for spores), and after 30 h, the average pellet diameter was around 1,000 μm, at a pH decreasing to about 3.0 in both cultures (Fig. S5).

When spores of both microorganisms were inoculated simultaneously (Fig. 2f), the growth curve and pH profile closely mirrored those of the axenic culture of *A. niger* (inoculated from spores). Similarly, when *S. coelicolor* spores were added to *A. niger* pellets (Fig. 2e), the co-culture showed growth characteristics resembling an *A. niger* axenic culture with pellet formation. This was likely because the fungus consistently outgrew the bacteria, which were opposed to acid stress in the resulting acidic conditions. *A. niger* usually grows well within a pH range of between 3.0 and 6.0, whereas *S. coelicolor* prefers a neutral to basic environment (pH > 5.0) [3, 39]. Observations from microscopic images (data not shown) also confirmed fungal outgrowth.

When *S. coelicolor* pellets replaced the spores in a co-culture with fungal spores, it was expected that *S. coelicolor* would dominate the culture (Fig. 2d). However, *A. niger* outgrew this cultivation again, this time in dispersed growth as verified with Gram-staining (data not shown). A sharp drop in pH indicated metabolic dominance. Reduced phosphate consumption relative to axenic *S. coelicolor* cultures (Fig. S3) might also indicate suppression of bacterial growth, as bacteria typically assimilate phosphate more rapidly than *A. niger*. Finally, when both microorganisms were inoculated in their pellet forms (Fig. 2g), overall growth was slower (µ = 0.12 h^− 1^). Although the fungus eventually dominated the culture, the higher phosphate uptake (Fig. S3) relative to an *A. niger* axenic culture suggests that *S. coelicolor* was also actively growing. This was further evident in microscopic images, with an increase in the pellet diameter of both microorganisms. As shown in Fig. S5, *S. coelicolor* pellets were found attached to those of *A. niger*, forming large aggregates where hyphal entanglement between the two species was visible (also shown in Fig. S6).

Accordingly, pellet-to-pellet inoculation was selected for all subsequent experiments, as it allowed the simultaneous growth of both microbial strains.

### Impact of glutamic acid availability

Although previous experiments showed that inoculating pellets led to a more balanced growth of both strains, *A. niger* pellets grew to a diameter larger than 3,000 μm on average, eventually dominating the smaller *S. coelicolor* pellets (average diameter of 275 μm), as shown in Fig. S5. Additionally, nitrogen limitation at 1.5 g L⁻¹ glutamic acid likely restricted *S. coelicolor* growth. Since nitrogen concentration influences *A. niger* morphology [31, 40, 41], we tested several initial glutamic acid concentrations of 0.5, 2.5, 5.0, and 10.0 g L⁻¹ to achieve a more homogeneous morphology and size distribution by reducing the fungal pellet size while preventing potential early nitrogen depletion.

**Fig. 3:**
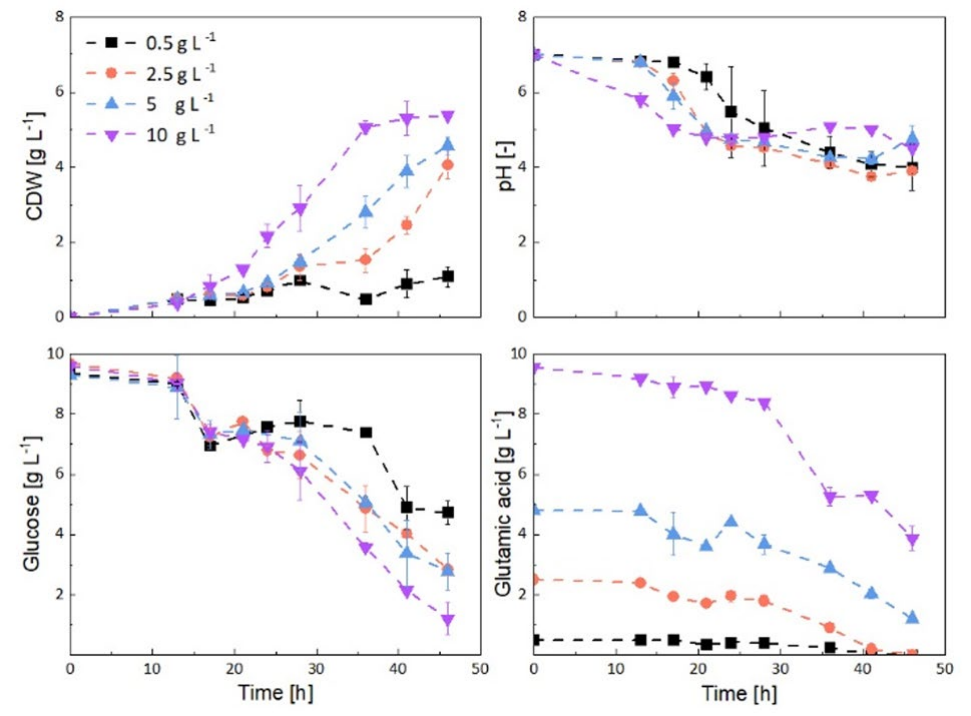
Effect of initial glutamic acid concentration (0.5, 2.5, 5.0, and 10 g L^-1^) on the growth, pH, glucose, and glutamic acid uptake of *Aspergillus niger* in axenic shake flask cultivations

Increasing the initial glutamic acid concentration from 2.5 to 10.0 g L^− 1^ reduced the duration of the lag phase by 5 h and consistently enhanced the average specific growth rate from 0.08 to 0.22 h^− 1^ (17 to 41 h) across independent experiments (Fig. 3). A correlation between the initial glutamic acid concentration and the subsequent biomass yield was observable with the highest biomass yield of 0.75 g g^− 1^ achieved at 10 g L^− 1^ initial glutamic acid concentration. During the exponential growth phase, the pH-value stabilized at approximately 5.0 with 10 g L^− 1^ glutamic acid, whereas it declined to approximately 4.0 during growth with 2.5 and 5.0 g L^− 1^ glutamic acid. This suggests that faster nitrogen limitation at lower glutamic acid concentrations leads to increased secretion of organic acids. Simultaneously, at higher glutamic acid concentrations, ammonia released during glutamic acid catabolism may have partially buffered the medium, thereby contributing to the higher pH.

As the initial glutamic acid concentration increased, the mean pellet diameter initially increased from 1,250 μm at 0.5 g L⁻¹ to 1,560 μm at 2.5 g L⁻¹ but then decreased to 1,390 μm at 5.0 g L⁻¹ and 988 μm at 10.0 g L⁻¹ (Fig. S7). Additionally, the number of pellets increased with higher glutamic acid concentrations. At 10 g L⁻¹, the pellets displayed a more homogeneous, well-defined size distribution (Fig. S7). This was confirmed by analyzing the standard deviation of pellet size and the normalized span width (Table S5), both of which showed a decreasing trend with increasing glutamic acid concentration. The standard deviation increased from 187 μm at 10 g L⁻¹ to 270 μm at 5.0 g L⁻¹, 282 μm at 2.5 g L⁻¹, and 312 μm at 0.5 g L⁻¹. Similarly, the normalized span width increased from 0.38 at 10 g L⁻¹ to 0.43 at 5.0 g L⁻¹, 0.44 at 2.5 g L⁻¹, and 0.54 at 0.5 g L⁻¹. This formation of smaller, more uniform pellets can provide a suitable starting point for co-cultivation experiments, as it maintains consistent conditions, generally minimizes mass-transfer limitations that are present in larger pellets, and increases the surface area for potential hyphal-hyphal interactions. Although cultivations with an initial glutamic acid concentration of 10 g L^− 1^ yielded the smallest pellets and the highest growth rate, the concentration never fell below 4.0 g L^− 1^, well above levels observed in the other cultivations, indicating that further increases in glutamic acid concentration would most likely have no effect. Therefore, 10 g L⁻¹ was selected as a suitable initial concentration for co-cultivation and used in all subsequent experiments.

**Table 1:** Impact of cultivation conditions on process parameters of axenic and co-cultures of *Aspergillus niger* and *Streptomyces coelicolor* in shake flasks

Experiment	Flask design (BF: baffled, NB: non-baffled)	µ [h^− 1^]	$\:{r}_{S\:Glc\:}$ [g (L h)^−1^]	$\:{q}_{S\:Glc\:}$ [g (g h)^−1^]	$\:{r}_{S\:Glu\:}$ [g (L h)^−1^]	$\:{q}_{S\:Glu\:}$ [g (g h)^−1^]	$\:{Y}_{X/S}*$ [g g^− 1^]
Axenic-culture: Inoculation workflow							
* A. niger* from spores	NB	0.10 ± 0.00	0.17 ± 0.01	0.12 ± 0.01	N/A	N/A	0.62 ± 0.02
* A. niger* from pellets (pre-culture)	NB	0.30 ± 0.00	0.59 ± 0.01	0.23 ± 0.01	N/A	N/A	0.67 ± 0.06
* S. coelicolor* from pellets (pre-culture)	NB	0.14 ± 0.00	0.29 ± 0.02	0.08 ± 0.00	N/A	N/A	0.39 ± 0.06
Co-culture: Inoculation workflow							
* A. niger* spores to *S. coelicolor* spores	NB	0.14 ± 0.05	0.17 ± 0.02	0.19 ± 0.01	N/A	N/A	0.54 ± 0.07
* A. niger* spores to *S. coelicolor* pellets	NB	0.11 ± 0.01	0.27 ± 0.07	0.06 ± 0.01	N/A	N/A	0.40 ± 0.07
* A. niger* pellets to *S. coelicolor* germinated spores	NB	0.36 ± 0.02	0.55 ± 0.01	0.23 ± 0.04	N/A		0.64 ± 0.05
* A. niger* pellets to *S. coelicolor* pellets	NB	0.12 ± 0.00	0.30 ± 0.00	0.10 ± 0.00	N/A	N/A	0.49 ± 0.00
Glutamic acid concentration [g L^− 1^]							
0.5	NB	0.03 ± 0.00	0.20 ± 0.02	0.39 ± 0.06	0.02	0.04	0.37 ± 0.08
2.5	NB	0.08 ± 0.00	0.19 ± 0.00	0.26 ± 0.04	0.02	0.09	0.57 ± 0.05
5.0	NB	0.14 ± 0.01	0.20 ± 0.01	0.17 ± 0.01	0.15	0.14	0.71 ± 0.02
10.0	NB	0.22 ± 0.01	0.24 ± 0.02	0.16 ± 0.03	0.26	0.07	0.75 ± 0.02
Pre-culture liquid volume/shear force							
50 mL pre-culture	NB	0.17 ± 0.01	0.42 ± 0.00	0.20 ± 0.02	0.21 ± 0.02	0.08 ± 0.01	0.51 ± 0.01
50 mL pre-culture	BF	0.17 ± 0.01	0.39 ± 0.05	0.15 ± 0.00	0.22 ± 0.01	0.09 ± 0.02	0.45 ± 0.05
75 mL pre-culture	NB	0.19 ± 0.00	0.48 ± 0.05	0.14 ± 0.02	0.21 ± 0.00	0.06 ± 0.00	0.47 ± 0.04
75 mL pre-culture	BF	0.24 ± 0.01	0.61 ± 0.12	0.21 ± 0.04	0.27 ± 0.00	0.08 ± 0.00	0.40 ± 0.00
Impact of shaking velocity [rpm]							
250	BF	0.16 ± 0.01	0.39 ± 0.03	0.15 ± 0.00	0.26 ± 0.00	0.10 ± 0.01	0.45 ± 0.05
136	BF	0.19 ± 0.00	0.30 ± 0.03	0.11 ± 0.01	0.21 ± 0.02	0.07 ± 0.02	0.65 ± 0.00
60	BF	0.03 ± 0.01	0.09 ± 0.03	0.04 ± 0.02	0.06 ± 0.00	0.03 ± 0.00	0.42 ± 0.01
Inoculation ratio (*A. niger*: *S. coelicolor*)							
1:1	NB	0.17 ± 0.01	0.38 ± 0.02	0.20 ± 0.02	0.20 ± 0.00	0.08 ± 0.01	0.51 ± 0.01
1:2	NB	0.15 ± 0.01	0.27 ± 0.01	0.12 ± 0.03	0.31 ± 0.01	0.16 ± 0.03	0.67 ± 0.03
1:5	NB	0.16 ± 0.00	0.24 ± 0.0	0.11 ± 0.02	0.25 ± 0.04	0.08 ± 0.00	0.69 ± 0.15

The process parameters are calculated as described in Table S7

*Yx/s refers to the biomass accumulation with reference to the consumed glucose and can therefore be above 0.5

### Impact of the shear force regime

**Fig. 4:**
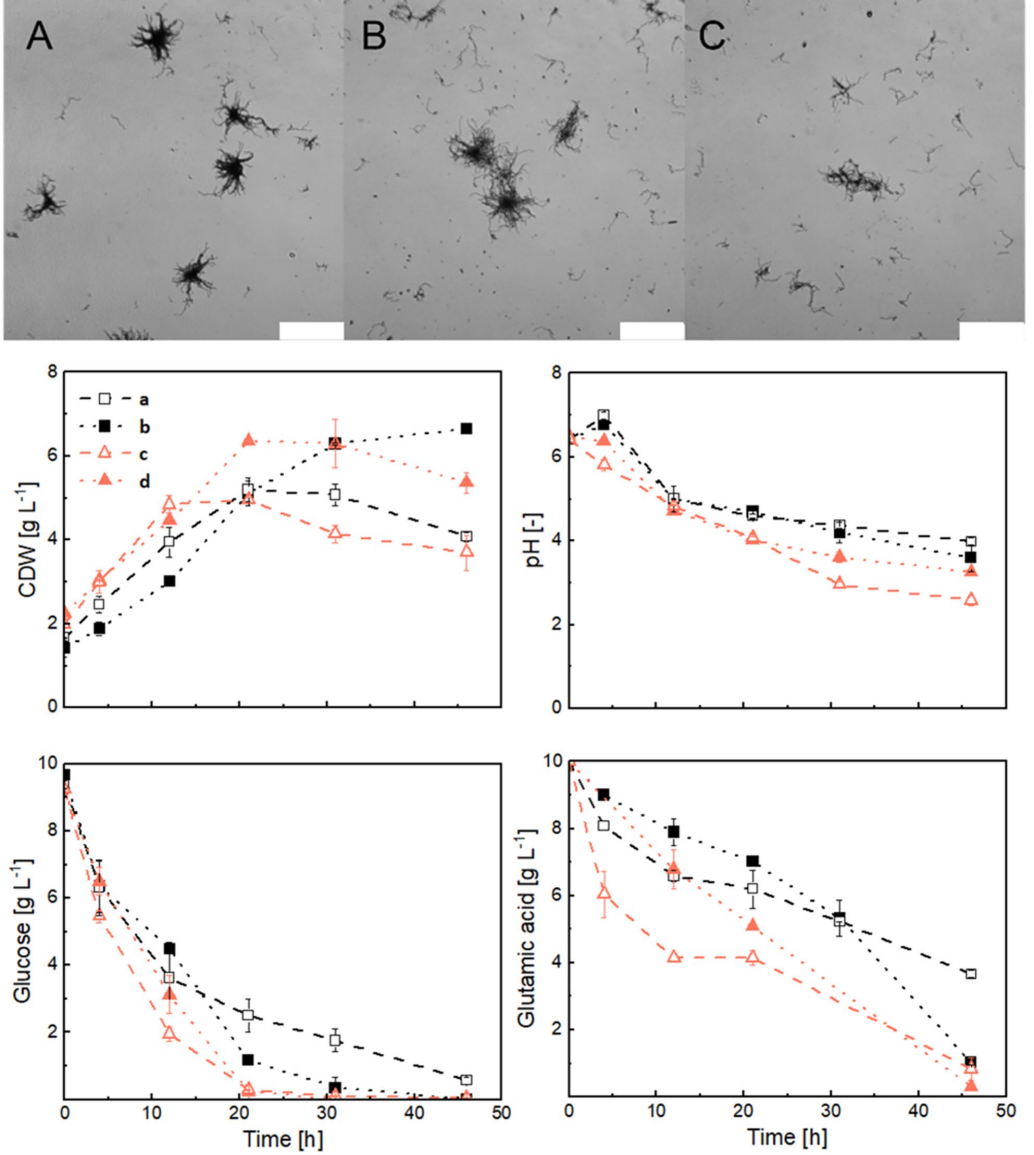
Top: Effect of medium volume on *A. niger* morphology in the pre-culture. A: 50 mL (6.25 × 105 spores mL^-1^), B: 75 mL (4.2 × 105 spores mL^-1^), C: 100 mL (3.2 × 105 spores mL^-1^). Images were taken at 12.5× magnification using a DIC microscope at 12 hours, the scale bar represents 1 mm. Bottom: Effect of different pre-cultivation conditions and shear forces (a: 50 mL pre-culture; baffled flask, b: 50 mL pre-culture; non-baffled flask, c: 75 mL pre-culture; baffled flask, d: 75 mL pre-culture; non-baffled flask) on growth, pH, and substrate consumption in *S. coelicolor* and *A. niger *co-culture (250 rpm, 1:1 inoculation ratio).

Using an initial glutamic acid concentration of 10 g L⁻¹ in the co-culture medium, the effects of pre-culture medium volume and shear force regime were then investigated, while keeping the total spore number and inoculation ratio constant. Specifically, three volumes of 50, 75, and 100 mL in NB pre-culture shake flasks (total volume of 500 mL) were initially tested. *S. coelicolor* pellet size and shape remained rather similar across all conditions (Fig. 4A–C). However, the size distribution of *A. niger* pellets was strongly affected. The dense pellet core with loose hyphae gradually got smaller as the volume increased from 50 to 100 mL, corresponding to a reduction in spore concentration (in the pre-culture). These morphologies were then used as inoculum for co-culture experiments, except for the 100 mL condition, which was excluded because of its dispersed morphology. Co-cultivation was performed using BF and NF to simultaneously investigate the impact of baffle-induced shear forces on growth characteristics and morphology. Figure 4 shows the growth, pH, and substrate consumption of co-culture experiments.

Specific growth rates were approximately 0.17 h⁻¹ with 50 mL pre-culture volume, both in BF and NF, and 0.19 h⁻¹ for 75 mL pre-culture volume in NF, while reaching 0.24 h⁻¹ in BF (0 to 30 h). Accordingly, the glucose and glutamate consumption rates were highest in cultures with 75 mL pre-culture volume in BF. This increase in substrate uptake and slightly shorter growth period observed with a 75 mL pre-culture volume may be attributed to both the looser pellet structure and the presence of baffles, which reduce mass transfer limitations within and around the pellets. The effect of baffles was particularly pronounced at this pre-culture volume. Additionally, the pH in both flask types of the 75 mL cultures was lower than in cultures with a 50 mL pre-culture volume.

**Fig. 5:**
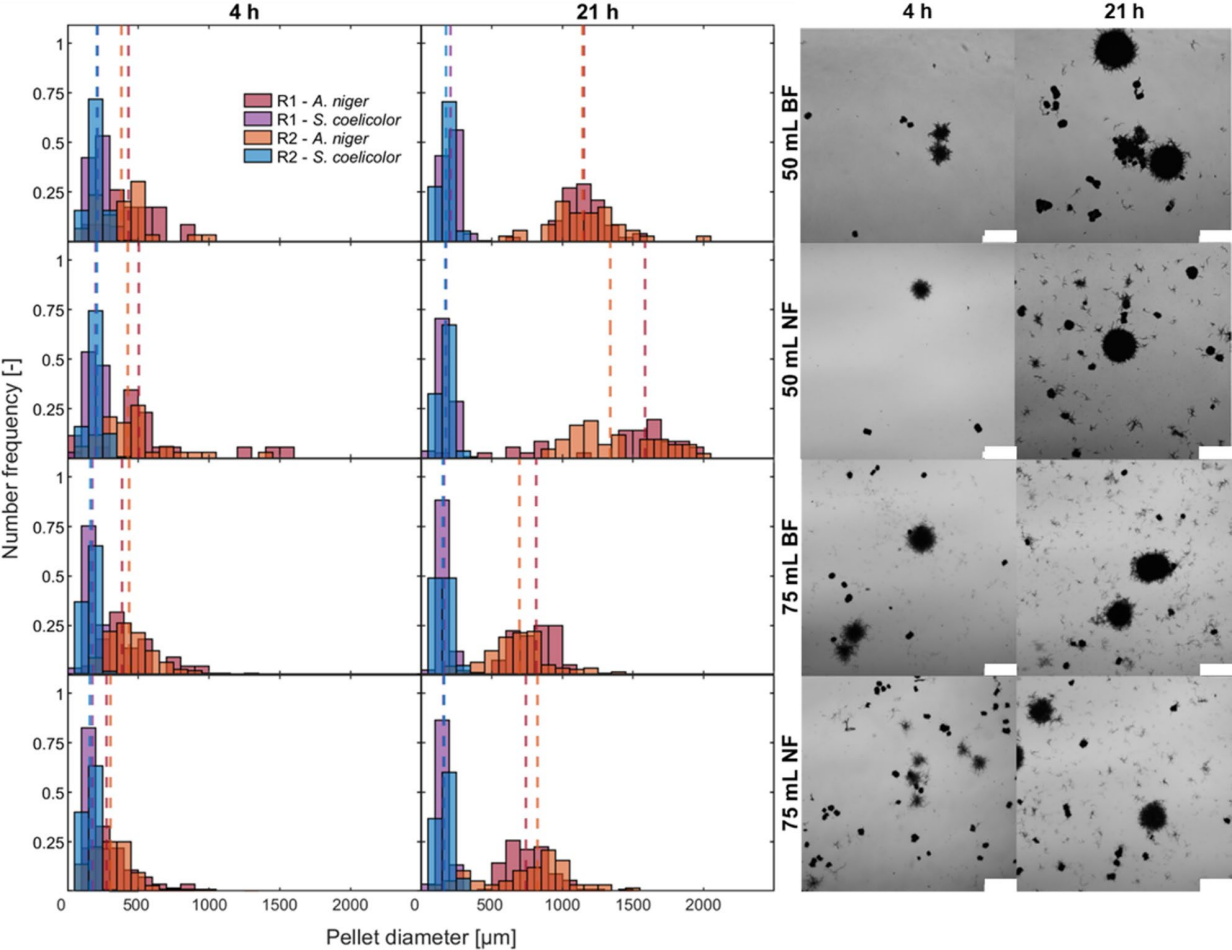
Left: Pellet size distribution of *Streptomyces coelicolor* and *Aspergillus niger* in co-cultivation experiments with baffled and unbaffled shake flasks starting from different pre-culture medium volumes. Data is presented as histogram plots (number frequency q_0_, bars) and D50 (median pellet diameter, lines). R1 and R2 are biological replicates. Right: Microscopic images of corresponding co-cultures. Images were taken at 12.5× magnification using a DIC microscope. The scale bar represents 1 mm (250 rpm, 1:1 inoculation ratio)

The initial mean pellet diameter of *S. coelicolor* was 180 ± 20 μm under all conditions (Fig. 5) and remained stable after 21 h. In contrast, *A. niger* pellets grew much larger, reaching 1,160 μm in BF and 1,350 μm in NF with 50 mL pre-culture. As shown in Fig. S8, entanglement of fungal and bacterial hyphae was also observed in this culture (BF with 50 mL). Increasing the pre-culture volume to 75 mL resulted in smaller final *A. niger* pellets (~ 800 μm) that were more homogeneous, likely due to initially smaller diameters; this faster fungal growth ultimately led to *A. niger* overgrowing the bacteria. Standard deviations for pellets with 75 mL volume, both BF and NF, were comparable to those in 50 mL. Bacterial pellets remained largely insensitive to baffles, with similar diameters under both conditions. Their relatively small size likely makes them less susceptible to shear forces, although their diameter decreased slightly (~ 15%) when the pre-culture volume increased from 50 mL to 75 mL. A two-way ANOVA followed by Tukey’s post-hoc test for the pellets at 21 h (Table S6) shows that the p-values for *A. niger* are consistently lower than those for *S. coelicolor*. The pre-culture volume had a significant effect on *A. niger* pellet size (*p* < 0.05), whereas the effect of baffles was weaker and not significant. The interaction between pre-culture volume and the presence of baffles was not significant, indicating that the two factors act independently and that the influence of pre-culture volume is independent of baffles.

Therefore, the flask type and pre-culture volume can influence *A. niger* growth and size distribution, with minimal impact on *S. coelicolor*. The 50 mL pre-culture volume can be favored over 75 mL for its slower fungal growth rate (facilitating the growth of *S. coelicolor* alongside *A. niger*) and the higher final pH. In this case, a BF would be more suitable than a NF due to the more homogeneous pellet diameter distribution.

**Fig. 6:**
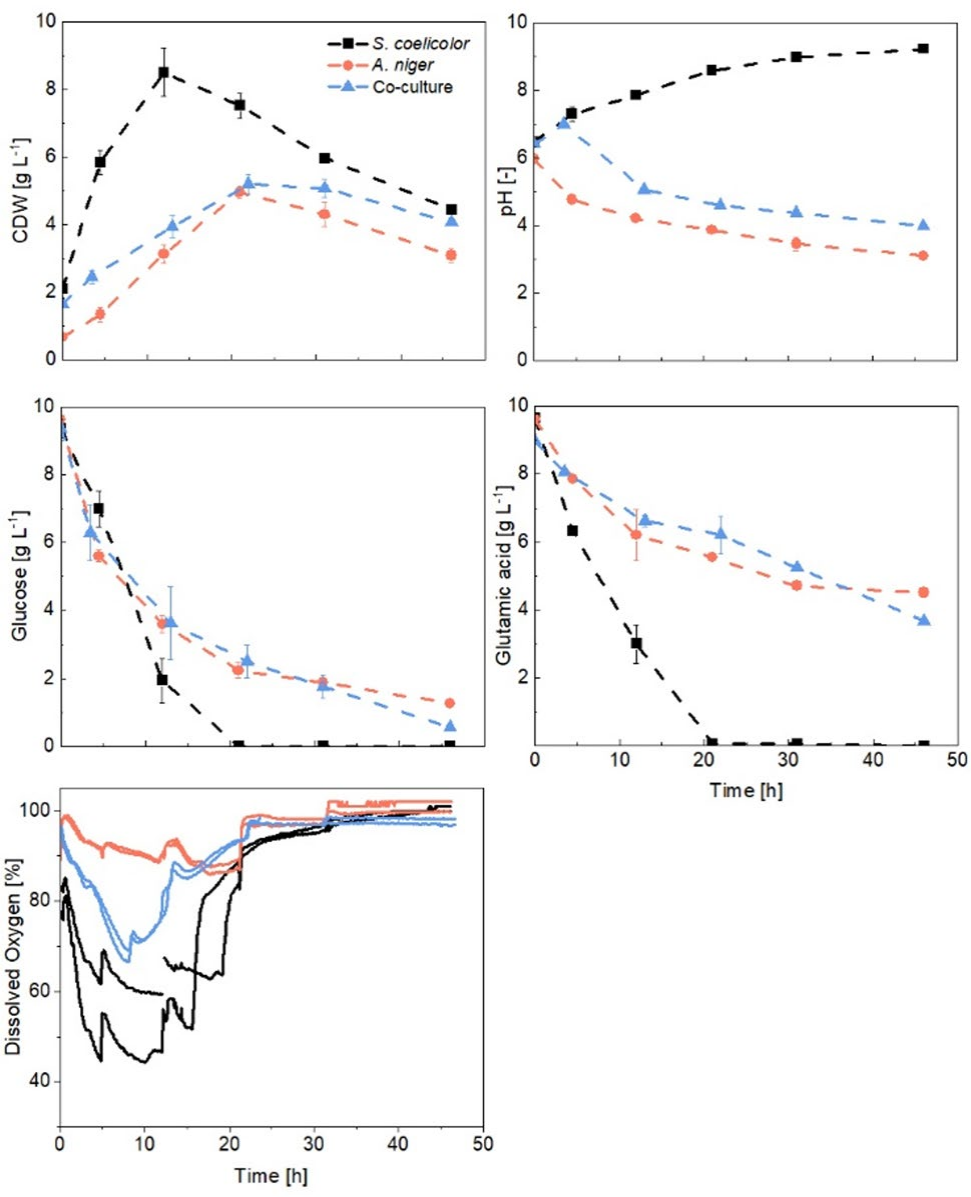
Comparison of dissolved oxygen [%], growth, pH, and substrate consumption in axenic and co-cultures of *Streptomyces coelicolor* and *Aspergillus niger* (with 50 mL pre-culture volume, 250 rpm, 1:1 inoculation ratio)

To provide a more comprehensive evaluation of the co-cultivation experiments, reference cultures of both *S. coelicolor* and *A. niger* were grown in axenic culture under conditions similar to co-cultures, with a 50 mL pre-culture medium volume. DO measurements were recorded in both axenic and co-culture conditions. Figure 6 presents the growth curves and pH, substrate, and DO concentrations of the axenic cultures compared with the reference co-culture (conducted in a BF with a 50 mL pre-culture volume).

Distinct growth rates were observed, with *S. coelicolor* at 0.36 h⁻¹ and *A. niger* at 0.21 h⁻¹. *A. niger* cultures had lower uptake rates (r_S Glc_ = 0.33 g L⁻¹h⁻¹ and r_S Glu_ = 0.18 g L⁻¹ h⁻¹) compared to *S. coelicolor* (r_S Glc_ = 0.56 g L⁻¹h⁻¹ and r_S Glu_ = 0.50 g L⁻¹h⁻¹). The co-culture performance resembled *A. niger* axenic culture, as expected due to its dominance in the co-cultivation. However, pH profiles might show that *S. coelicolor* was actively growing during the first four hours of cultivation. The consistently higher pH in the co-culture, compared to values below 4.0 in *A. niger*’s axenic culture, also might indicate that *S. coelicolor* remains metabolically active and influences the overall cultivation process. During exponential growth, the DO declines more sharply in *S. coelicolor* than in *A. niger*, with co-culture showing an intermediate slope. The DO concentration decreased to only about 85% in the fungal culture and remained high throughout the cultivation, suggesting that oxygen stress may have influenced the termination of the cultivation process. At sampling times, the measurement is disrupted, and DO concentration is affected, likely due to reduced medium volume and increased k_L_a values thereafter.

Pellet size distributions for axenic cultures are shown in Fig. S9. Mean pellet diameter of both partners increased until 21 h, but *A. niger* pellets grew larger in co-culture than in axenic cultures, while the opposite was observable for *S. coelicolor*.

### Impact of power input

**Fig. 7:**
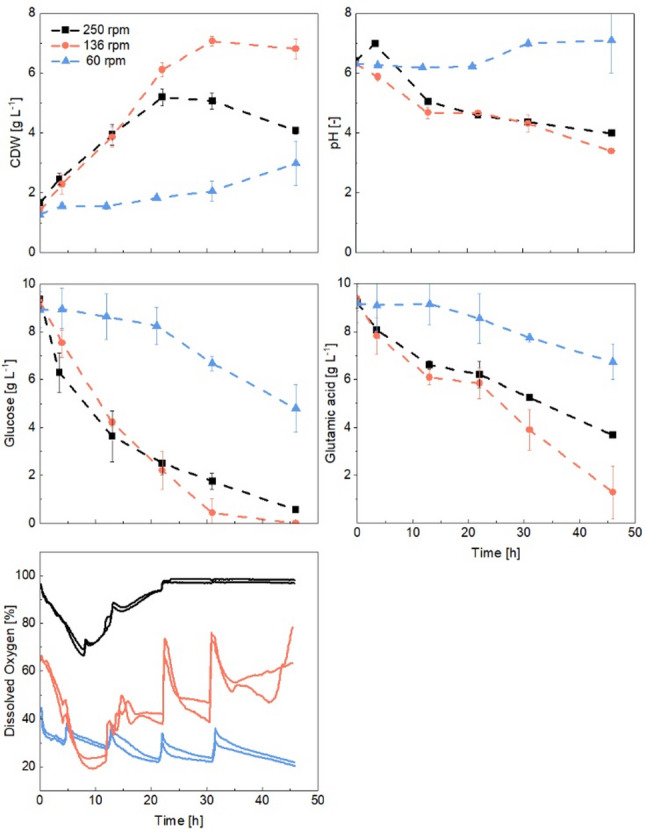
Effect of different shaking velocities (250, 136, and 60 rpm) on the dissolved oxygen [%], growth, pH, and substrate consumption in co-cultures of *Streptomyces coelicolor* and *Aspergillus niger* (with 50 mL pre-culture volume, 1:1 inoculation ratio)

To investigate whether oxidative stress (at hyphae at the outer region of pellets) influences the co-culture, the shaking frequency was decreased in some cultures, reducing the k_L_a value and reaching about 25% (136 rpm) and 1% (60 rpm) of the maximum OTR in comparison to the previously applied 250 rpm. This led to initial dissolved oxygen concentrations of 100% with 250 rpm, 66% with 136 rpm, and 45% with 60 rpm. Figure 7 shows the growth patterns, pH changes, substrate concentrations, and the dissolved oxygen profile under these conditions. In the case of DO, as previously discussed, the measurement is shortly disturbed at the sampling points (at 4, 8, 12, 22, and 31 h).

At 136 rpm, the co-culture achieved a higher maximum CDW of 7.07 g L⁻¹ compared to 250 rpm, resulting in a higher glucose-specific biomass yield (Y_x/s_ = 0.65 g g⁻¹ vs. 0.45 g g⁻¹ at 250 rpm). This potential metabolic shift could have supported *S. coelicolor* growth; however, the lack of an initial pH increase indicates that *A. niger* likely dominated the early growth phase. Further reducing the shaking speed to 60 rpm caused a lag phase of 13 h, leading to the lowest growth rate (µ = 0.03 h⁻¹), as well as the lowest substrate consumption rates (0.09 g (L h)^−1^ for glucose, 0.06 g (L h)^−1^ for glutamate). Interestingly, the pH exceeded 7.0, possibly suggesting the eventual dominance of *S. coelicolor* under these low shear force and OTR conditions.

**Fig. 8:**
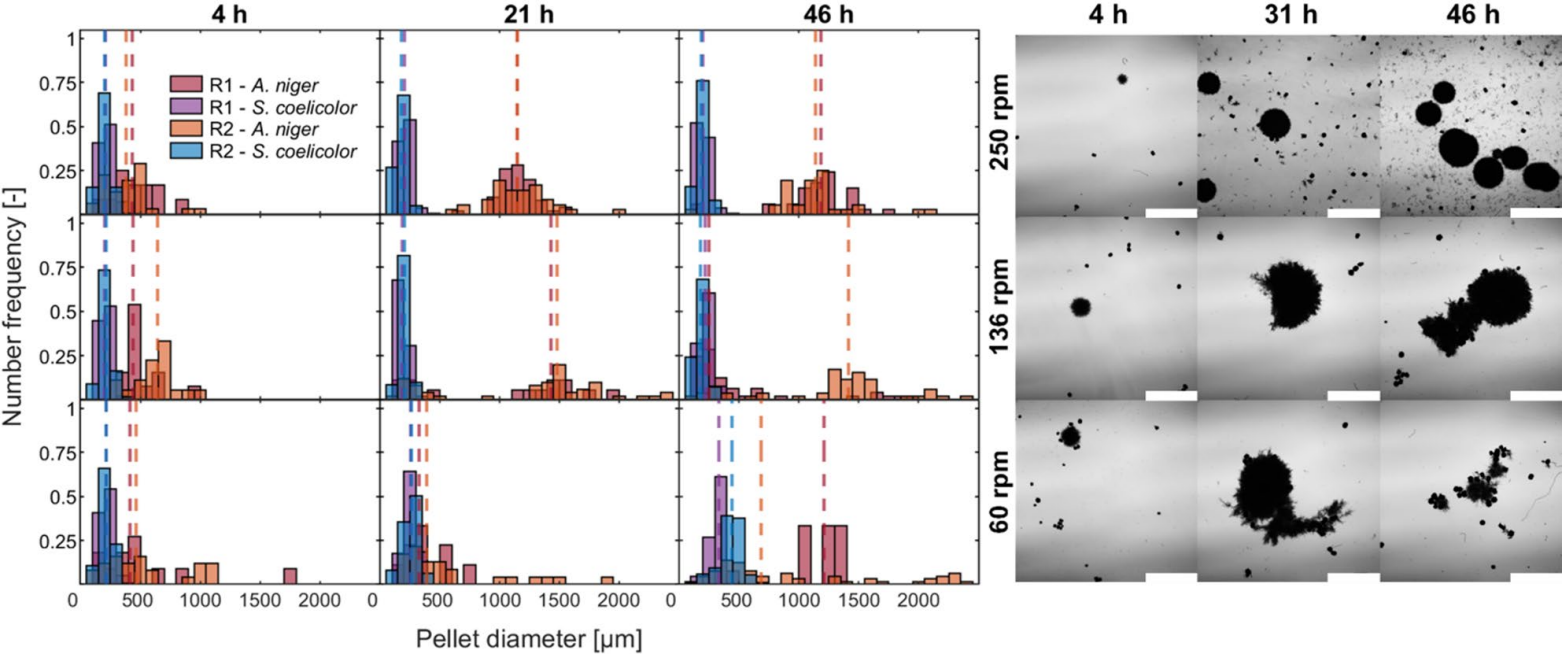
Left: Pellet size distribution of *Streptomyces coelicolor* and *Aspergillus niger* in co-cultivation experiments at 60, 136, and 250 rpm shaking velocities in baffled shake flasks. Data is presented as histogram plots (number frequency q_0_, bars) and D50 (median pellet diameter, lines). R1 and R2 are biological replicates. Right: Microscopic images of corresponding co-cultures. Images were taken at 12.5× magnification using a DIC microscope, the scale bar represents 1 mm (with 50 mL pre-culture volume, 1:1 inoculation ratio)

Although the lowest shaking frequency did not cause oxygen limitation, the reduced velocity and OTR negatively impacted *A. niger* growth. As shown in the histograms in Fig. 8, after 21 h at 136 rpm, 90% of *A. niger* pellets exceeded a diameter of 1,200 μm, while the pellet size of *S. coelicolor* remained largely unchanged. These morphological changes corresponded with the observed pH variations.

The influence of the shaking velocity was significant (*p* < 0.05, Table S6). The standard deviation for pellet size in *A. niger* increased over time across all shaking frequencies, where cultures became more heterogeneous as cultivation progressed (Table S5). At lower shaking frequencies (particularly 60 rpm), the standard deviation and normalized span width showed significant variations among replicates, reflecting poor reproducibility. Additionally, *A. niger* formed large clumps and various aggregates at 60 rpm (Fig. S10), likely due to insufficient shear forces, which hindered uniform pellet growth and promoted clump formation. As a result, not all pellet diameters could be accurately measured, leading to an underestimation of the heterogeneity. In contrast, at 250 rpm, the span width remained relatively stable over time, as the higher shaking frequencies promoted a more homogeneous and reproducible culture. For *S. coelicolor*, on the other hand, a more homogeneous size distribution was observed, with a much lower standard deviation throughout the cultivation, regardless of the shaking frequency.

In summary, shaking frequency (power input) played a crucial role in influencing pellet size distribution and growth by modulating oxygen transfer rates and shear force. At 250 rpm, *A. niger* grew more homogeneously, demonstrating robustness to higher shear. In contrast, *S. coelicolor* grew better at lower shaking frequencies (60 and 136 rpm). This could possibly be due to sensitivity to higher shear forces and/or oxidative stress.

### Impact of inoculating ratio

**Fig. 9:**
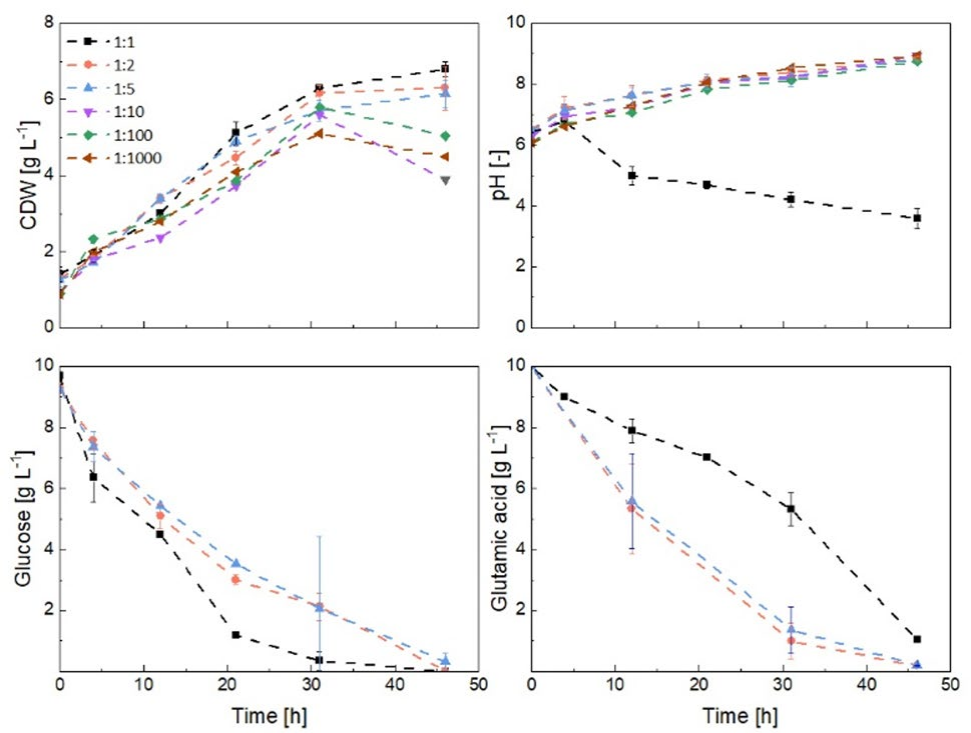
Effect of varying the inoculation ratio of *Aspergillus niger* to *Streptomyces coelicolor* spores (1:1, 1:2, 1:5, 1:10, 1:100, 1:1,000) on growth, pH, and substrate consumption in co-cultivation. Substrate concentrations were measured only for inoculation ratios of 1:1, 1:2 and 1:5 (250 rpm and 50 mL pre-culture volume)

At the final stage, the impact of different inoculation ratios of *A. niger* to *S. coelicolor* (pellet to pellet) was investigated to promote *S. coelicolor* growth. Shake flask experiments were conducted using inoculation ratios of 1:1, 1:2, 1:5, 1:10, 1:100, and 1:1,000 (*A. niger* to *S. coelicolor*). To achieve various ratios, *S. coelicolor* spore concentration was kept constant at 5 × 10⁶ spores mL^− 1^ in the pre-culture, while *A. niger* concentration (6.25 × 10⁵ spores mL^− 1^) was adjusted accordingly. Figure 9 illustrates the growth patterns, pH changes, and substrate concentrations throughout the co-cultivations.

**Fig. 10:**
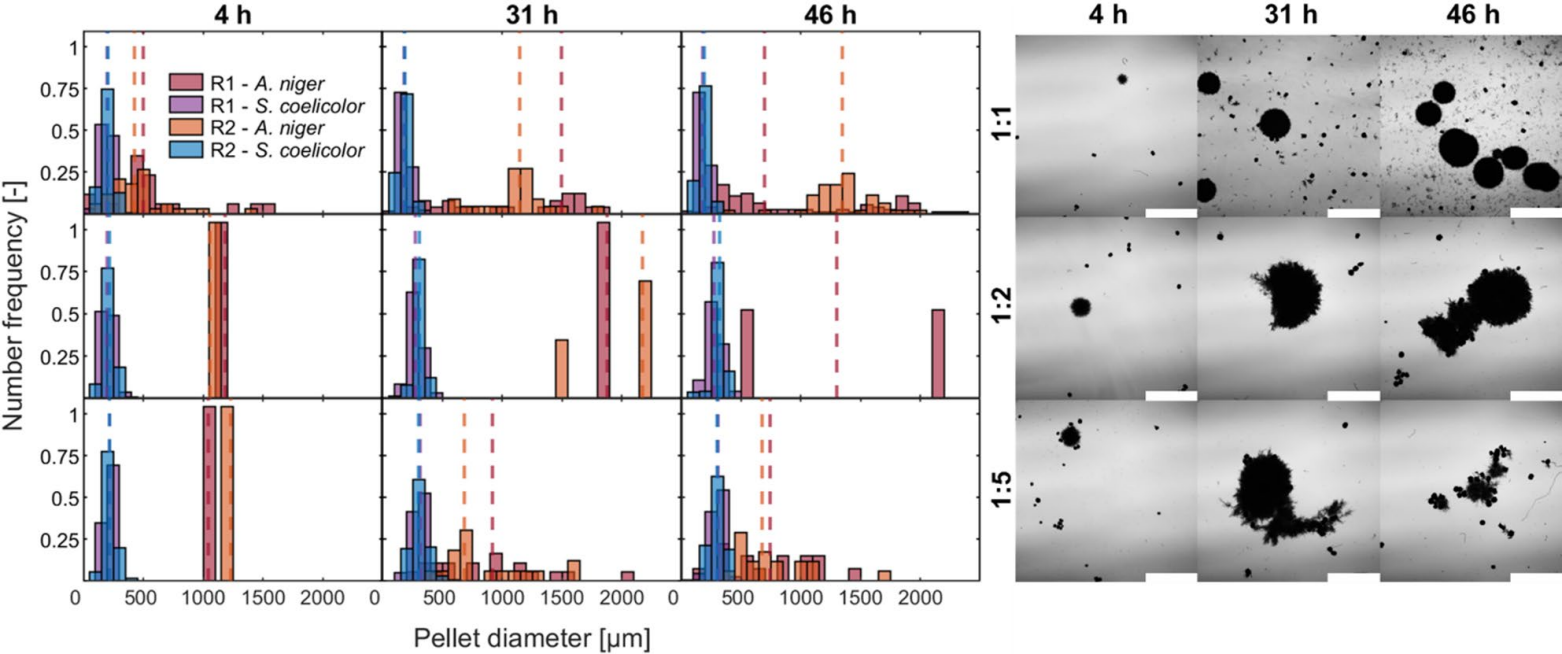
Left: Pellet size distribution of *Streptomyces coelicolor* and *Aspergillus niger* in co-cultivation experiments at inoculation ratios of 1:1, 1:2, and 1:5 (fungal to bacteria). Data is presented as histogram plots (number frequency q_0_, bars) and D50 (median pellet diameter, lines). R1 and R2 are biological replicates. Right: Microscopic images of corresponding co-cultures. Images were taken at 12.5× magnification using a DIC microscope. The scale bar represents 1 mm (250 rpm and 50 mL pre-culture volume)

Despite rather similar growth patterns in all the cultures (Table 1), various pH-values and substrate consumption profiles were observed. In all experiments, the pH-value initially increased within the first 4 h, suggesting that *S. coelicolor* started to consume glutamic acid instantly. This trend continued for all the conditions except for 1:1 inoculation ratio. In this culture, the pH subsequently dropped below 5.0, signaling a shift toward *A. niger* dominance. Notably, glutamic acid consumption rates increased at higher *S. coelicolor* inoculation levels, increasing from 0.19 g h⁻¹ at the 1:1 ratio to 0.31 and 0.25 g h⁻¹ at 1:2 and 1:5 ratios, respectively. On the contrary, glucose uptake rate was higher in 1:1 (0.38 g L^− 1^) than in the other two ratios (0.27 and 0.24 g L^− 1^ for 1:2 and 1:5, respectively). Automated image analysis was conducted only on samples from cultures inoculated at 1:1, 1:2, and 1:5 ratios, as the remaining conditions lacked sufficient *A. niger* pellet numbers for statistically meaningful analysis. After 4 h, *S. coelicolor* pellets had a mean diameter of approximately 200 μm. In contrast, *A. niger* formed markedly larger pellets, with a mean diameter of approximately 500 μm at a 1:1 ratio and 1,000 μm at 1:2 and 1:5 ratios (Fig. 10). By 31 h (the end of the exponential growth phase), *S. coelicolor* pellets maintained their size at 1:1 but grew to a mean diameter of around 300 μm at the 1:2 and 1:5 ratios. This increase could suggest a growth advantage of *S. coelicolor* consistent with the observed pH profile.

Bacterial pellets maintained a homogeneous, reproducible pellet size distribution with a standard deviation of approximately 50 μm at a constant span width (1:1 inoculation ratio). *A. niger* pellets were more heterogeneous compared to *S. coelicolor* (1:1 inoculation ratio), increasing over time (Table S5). At a 1:2 inoculation ratio, the lower concentration of fungal spores in the pre-culture resulted in larger, fewer pellets, as observed in microscopic images, and insufficient pellet counts for statistical analysis at certain time points. At a ratio of 1:5, more loose hyphae and pellet fragments were observed, which are probably due to the breakage of the pellets. A one-way ANOVA performed on pellet data at 31 h (Table S6) revealed that the inoculation ratio exerted a greater influence on the *S. coelicolor* morphology than on *A. niger*.

In summary, increasing the inoculation ratio in favor of the bacteria (1:2 and 1:5) enhanced their competitive advantage, as indicated by the rise in pH and the increase in the pellet diameter. Owing to their inherently smaller pellet size, *S. coelicolor* populations grew more homogeneously than *A. niger*.

Although it was not the primary aim of this study to quantify secondary metabolites, kojic acid (5-hydroxy-2-hydroxymethyl-δ-pyrone) a fungal secondary metabolite that acts as an antibacterial and anti-inflammatory agent, was measured. Its action is based on cell integrity damage and has been examined at several bacterial strains [42]. It was analyzed as an illustrative example at the beginning of the stationary phase in all experiments. Kojic acid was detected in only two cultures, namely those with *A. niger* to *S. coelicolor* inoculation ratios of 1:2 and 1:5, with concentrations of 4.06 mg L⁻¹ and 3.17 mg L⁻¹, respectively. These were experiments with a remarkable growth of *S. coelicolor* in co-cultivation. Kojic acid is reported to be synthesized through the direct conversion of glucose via oxidation and dehydration [43, 44]. Not much is known so far about kojic acid synthesis in *A. niger*, while *Aspergillus oryzae* and *Aspergillus flavus* are known producers [43]. They accumulate kojic acid to more than 100 g L^− 1^ in submerged cultivation with glucose as the main carbon source [45]. It is interesting to note that so far, no report has been found about the stimulation of kojic acid synthesis by fungal-bacterial co-cultures.

## Discussion

In co-culture systems, controlling the population dynamics to achieve balanced growth of the involved organisms is essential for ensuring reproducibility while enabling interspecies interactions, as early dominance of one organism may shift the system toward monoculture-like behavior, ultimately affecting metabolic outcomes. Here, image analysis was selected as a suitable method to monitor the growth of *A. niger* and *S. coelicolor* in shake flask co-cultivation experiments. Overall, the image analysis results demonstrate that the proposed workflow enables reliable distinction and quantitative analysis of morphological features of both species in the co-culture.

At early cultivation time points, the morphological similarity between the two species limited the achievable classification accuracy to approximately 88%. As cultivation progressed and the morphological differences became more significant, accuracy increased accordingly (to 97%). However, under certain conditions, classification accuracy slightly decreased. This occurred, for example, when *A. niger* pellets became highly dispersed, making it difficult to delineate boundaries between closely adjacent fungal and bacterial structures, and in one case due to sample-handling issues, where insufficient pellet dispersion on the Petri dish hindered segmentation and classification. To further improve the method, incorporating texture-based descriptors, such as gray-scale value variance, local entropy or other pixel-level statistics, may better capture texture differences and thus enhance the robustness of classification across diverse cultivation conditions.

To establish an inoculation and cultivation workflow, *A. niger* and *S. coelicolor* were inoculated to form a co-culture either as spores or pellets from pre-cultures (12 h for *A. niger*, 20 h for *S. coelicolor*). When *S. coelicolor* was inoculated as spores, it failed to grow in parallel with *A. niger*, regardless of whether fungal spores or pellets were inoculated. This could be because, since *A. niger* spores agglomerate and form pellets earlier than *Streptomyces*, some bacterial spores may become trapped within the fungal pellet cores and, therefore, not have enough opportunities to develop into pellets after maturation [29, 30]. A similar phenomenon was reported by Boruta et al. when *Streptomyces rimosus* spores were outgrown by the competing fungus *Aspergillus terreus* in 5.5 L stirred tank reactor cultivations [6]. Apart from that, the metabolic side product secretion of *A. niger* reduces the pH-value to about 3.0, creating unfavorable growth conditions for *S. coelicolor* germinated spores, which do not efficiently grow at pH-values below 4.5-5.0 [3, 39]. Interestingly, the pH of some co-cultures dropped even lower than that of the *A. niger* axenic culture, suggesting a potential competitive strategy by *A. niger*, which may have intensified the secretion of acidic metabolites to suppress the bacteria [7].

In our study, *A. niger* grew as dispersed hyphae when bacterial pellets were inoculated with fungal spores. We assumed that bacterial pellets might have prevented fungal spore aggregation and pellet formation. In both cases, *A. niger* dominated the culture and suppressed bacterial growth. It was only in pellet-to-pellet inoculation that *S. coelicolor* could grow competitively. Although this culture could promote the prolonged growth phase of both protagonists, *A. niger*, as the faster-growing species, eventually overgrew *S. coelicolor*. Another study with similar microorganisms [3] reported the successful application of delayed inoculation, where *A. niger* spores were inoculated into the culture flasks 72 h after the inoculation of *S. coelicolor*. However, in our study, to reduce the abundance of any unknown side products in the co-culture medium, pellets of the pre-culture were washed and inoculated simultaneously in fresh medium.

Increasing the pre-culture volume led to changes in *A. niger* pellet morphology, notably a loss of hyphal density and pellet structure, which was qualitatively observed. Higher spore concentrations, which increase collision probability [46], may explain the denser pellets observed with the pre-culture volume of 50 mL. More importantly, previous research has shown that the filling volume in shake flasks significantly affects oxygen concentration and, consequently, the k_L_a-value. For example, in 250 mL NF with 50 to 125 mL of culture medium, the k_L_a-value decreased from 16.6 to 7.1 h⁻¹ at 140 rpm as the medium volume increased [47]. Similarly, in 250 mL NF at 250 rpm with a shaking diameter of 5 cm, the k_L_a decreased from 110 to 65 h⁻¹ as the filling volume increased from 25 to 40 mL [48]. Such reductions in oxygen concentration have been shown to decrease pellet density and increase mycelial growth in filamentous microbial systems such as *Streptomyces avermitilis* [49]. Since the co-culture medium volume remained constant, the faster growth of *A. niger* and its rapid dominance, indicated by a pH-value drop to 3.0, can be attributed to the smaller, less dense pellets formed in the pre-culture of 75 mL (BF).

As a crucial parameter, shaking frequency was subsequently changed to investigate whether a reduced OTR influences population dynamics of the co-culture. At 136 rpm, similar to 250 rpm, a decrease in pH indicated an eventual dominance by *A. niger*. Conversely, at 60 rpm, an increase in *S. coelicolor* pellet size and an initial pH rise suggested a shift favoring bacterial dominance. Schiefelbein et al. estimated the k_L_a values of 7, 120, and 280 h⁻¹ at 50, 150, and 250 rpm, respectively (compared to 60, 136, and 250 rpm in this study). 500 mL PreSens flasks (similar to this study) with a working volume of 50 mL were used in their experiment [34].

However, before concluding that these observations are solely due to decreased OTRs, the impact of shear forces has to be considered. Liu et al. demonstrated that reducing shaking frequency in 500 mL BF with a 100 mL working volume from 220 to 120 rpm decreased the k_L_a from 250 to 100 h⁻¹ and the shear force from 3.2 to 0.75 Pa [50]. Therefore, the reduced shear force and k_L_a at 136 rpm explain the extended growth period. The formation of the heterogeneous clumps of *A. niger* in our system at 60 rpm is, however, more likely to originate from reduced shear forces. Even though the k_L_a-value was significantly lower at 60 rpm (estimated at approx. 3.4 h⁻¹), it was still insufficient to induce oxygen-limited conditions in the media. Shear forces are known to be crucial to achieve a more homogeneous pellet-size distribution [51]. A previous study on *A. niger* showed that lower shaking frequency of 50-100 rpm led to formation of one large clump, while higher frequencies (150 rpm and above) resulted in the development of a population of smaller pellets [52]. The breakage of *Aspergillus niger* pellets under elevated hydrodynamic stress has also been discussed in detail in previous studies [53, 54].

We also observed higher reproducibility and reduced morphological heterogeneity when using baffled flasks. A single large clump, as in unbaffled flasks, is difficult to analyze anyway. To our knowledge, there is only one similar study that described this effect in a co-cultivation of *S. coelicolor* and *T. reesei* in 48-well plates [9]. The higher specific power input when increasing the shaking frequency from 800 to 1,200 rpm exerted a growth disadvantage on *S. coelicolor.* To promote higher bacterial growth in our study, reducing the fungal concentration proved essential, as *A. niger* was dominant under most of the conditions with equal inoculation ratios. Adjusting the inoculation ratio to 1:2 led to an increased mean pellet diameter for both strains, indicating biomass accumulation and allowing both populations to co-exist and grow simultaneously. Further reducing *A. niger* concentration at an inoculation ratio of 1:5 allowed *S. coelicolor* to thrive but led to degradation of *A. niger* biomass at later phases. Throughout some co-culture experiments (inoculation ratios of 1:2, 1:5), notable alterations in metabolic and pH profiles were observed, which could be attributed to the metabolic dominance of *S. coelicolor*. This was evidenced by an increase in pH because of glutamic acid uptake, aligning with our finding that pH shifts serve as a reliable indicator of the dominant species. The distinct pH preferences of the two species, as observed in their axenic culture, further supported this observation: under higher inoculation ratios in favor of *S. coelicolor*, the bacteria were able to outcompete *A. niger* as the pH inhibitory effect was mitigated. However, this does not necessarily mean that *A. niger* was no longer active, since the fungal secondary metabolite kojic acid was detected only in these two co-cultures, a probable agent to reduce bacterial growth.

Inoculation ratios of filamentous microorganisms have been investigated only in a few studies [29]. For instance, Finger et al. explored a co-culture system of *Trichoderma reesei* and *S. coelicolor* in 48-well plates (1 mL working volume, 800 rpm) under nutrient limitation [9]. They found that reducing the fast-growing *T. reesei* from an inoculation ratio of 0.1:1 to 0.001:1 led to decreased nutrient uptake and biomass formation of it, while both parameters increased for *S. coelicolor*. In our study, we hypothesize that these changes can be caused by a dynamic growth environment that may affect each strain differently. Additionally, different inoculation ratios may lead to distinct secondary metabolite profiles, as reported by Boruta et al. in a co-culture of *A. terreus* and *S. rimosus* [28]. Using NF with a working volume of 500 mL, they inoculated 200 mL of fresh medium with varying amounts of both microorganisms. The authors reported the highest secondary metabolite production at inoculation ratios of 6:14 to 12:8 (*A. terreus* to *S. rimosus*). Interestingly, even when one strain was less dominant, it could still induce secondary metabolite production in the co-culture [3]. Further metabolite analysis in our system would be necessary to explore such possibilities. It would also be interesting to narrow down the inoculation ratio range between 1:1 and 1:2 in future experiments. The kojic acid levels observed at 1:2 and 1:5 inoculation ratios are likely linked to both biological interactions and morphological changes. On one hand, kojic acid produced by *A. niger* may contribute to competitive inhibition of *S. coelicolor*. On the other hand, higher oxygen availability is known to enhance its synthesis [43]. The looser pellet morphology at high inoculation ratios likely improves internal oxygen transfer and a lower fraction of *A. niger* biomass may reduce overall oxygen consumption, further increasing oxygen availability.

It is worth mentioning that varying the inoculation ratios in the pre-culture without affecting *A. niger* pellet size proved challenging. We inoculated each co-cultivation flask using the entire biomass from a single pre-culture shake flask. To achieve a lower concentration of *A. niger* in the co-culture, we reduced the spore concentration in the pre-culture. Adjusting the medium volume was not feasible due to the impractically small volumes. Hence, this would have led to completely different fluid dynamics and morphology. While some studies, like Boruta et al. [28], used manual pipetting to transfer pellets from the pre-culture, this method could be prone to non-reproducible results, especially when larger pellets are involved. Instead, we focused on controlling spore concentrations in the pre-culture to adjust inoculation ratios. Our strategy could provide an assessment of how inoculation strategies influence pellet size and thereby microbial dominance.

Attachment of *S. coelicolor* pellets to *A. niger* was observed in some of the cultures, particularly at a 1:5 inoculation ratio (suggesting the role of physical contact in co-cultivation. A similar phenomenon was reported by [3] with the same strains in NF at 1:10 inoculation ratio. *S. coelicolor* is known to produce chitin-degrading proteins that bind to *A. niger* hyphae, using chitin as a carbon and nitrogen source [55, 56]. Therefore, nutrient limitation may have triggered the attachment in our study and could also explain the presence of *A. niger* dispersed mycelium in the medium.

Generally, in co-cultures, a producer-inducer dynamic typically applies: one protagonist primarily acts as an inducer with stimulatory activity, while the other takes on the role of a metabolite producer, requiring sufficient biomass development [10]. Strategies for controlling population dynamics should be tailored to the co-cultivation objective and the designated roles of the producer and inducer. An example according to our results would be: when *A. niger* is preferred as the producer, common in actinomycetes-fungi interactions [4], higher shaking frequency (250 rpm) and the use of baffles could possibly promote more reproducible and homogeneous fungal pellet size distribution. However, at a moderate shaking frequency of 136 rpm, the fungus would accumulate more biomass. Conversely, if the goal is to prioritize metabolite production by *S. coelicolor*, higher inoculation rates of the bacteria combined with lower power input could be more effective. For scenarios where both strains are required to act as producers, controlling bacterial growth becomes critical to ensure a balanced co-cultivation, as demonstrated at an inoculation ratio of 1:2 (bacteria to fungus).

## Conclusion

By employing a pellet-to-pellet inoculation method, stable co-cultivation of *A. niger* and *S. coelicolor* was achieved, enabling both microorganisms to grow simultaneously. However, the fungus consistently maintained a growth advantage under most conditions. Adjusting inoculation ratios proved a key parameter for modulating microbial dominance, with a 1:2 fungal-to-bacterial ratio supporting balanced growth of both, while other ratios favored either fungal or bacterial dominance. Power input (and thus oxygen availability) and shear forces, as varied by shaking frequency and flask design (baffled/non-baffled), played an important role in shaping both macromorphology and reproducibility. A higher shaking frequency (250 rpm) led to a uniform *A. niger* pellet population, whereas lower shaking frequencies (60 rpm) promoted bacterial growth and heterogeneous fungal size. Meanwhile, the pH-value served as a reliable indicator of species dominance, reflecting the interplay between microbial metabolic activities. While competitive interactions (e.g., bacterial hyphal attachment, pellet degradation) were observed under nutrient limitation, effective competition by *S. coelicolor* coincided with the onset of kojic acid production by *A. niger*.

By monitoring the morphological changes quantitatively, we gained a deeper understanding of how the less dominant organism still grows and competes, as evidenced by shifts in pellet diameter. This quantitative approach, rarely discussed in co-culture studies, proved essential for evaluating microbial interactions in this study. Future studies should integrate molecular-level analyses, such as transcriptomics or proteomics, to connect morphology patterns with physiological responses.

## Supplementary Information

Below is the link to the electronic supplementary material.

Supplementary Material 1.

## Data Availability

The datasets of the current study are available from the corresponding authors on reasonable request.

## List of symbols

µ [h^− 1^]: Specific growth rate
c* [mol L^− 1^]: Maximum dissolved oxygen concentration
CDW [g L^− 1^]: Cell dry weight
d [mm]: Maximum shake flask diameter
d_0_ [cm]: Shaking diameter
k_L_a [h^− 1^]: Volumetric gas-mass transfer coefficient
Osmol [Osmol kg^− 1^]: Osmolality
OTR [mol (L h)^−1^]: Oxygen transfer rate
p: Probability value
p_R_ [bar]: Working pressure
q_S Glc_ [g (g h)^−1^]: Specific glucose uptake rate
rpm [min^− 1^]: Revolution per minute
r_S Glc_ [g (L h)^−1^]: Volumetric glucose uptake rate
r_S Glu_ [g (L h)^−1^]: Volumetric glutamic acid uptake rate
V_L_ [mL]: Filling volume
V_max_ [mL]: Flask volume
y_O2_: Molar fraction of O_2_ in the gas phase
Y_X/S_: Substrate-related biomass yield

## Abbreviations

2× YT: Double-strength yeast tryptone medium
BF: Baffled flask
CM: Complete medium
D50: Median pellet diameter
DIC: Differential interference contrast microscope
DO: Dissolved oxygen
NF: Non-baffled flask
OTR: Oxygen transfer rate
SFM: Soy flour mannitol
SSBM-P: Phosphate-limited medium
