# Supplementary material for: Co-cultivation dynamics of the filamentous microorganisms Aspergillus niger and Streptomyces coelicolor in shake flasks
Source: Microb Cell Fact. 2026 May 6;25:122. doi: 10.1186/s12934-026-03015-9 (PMC13154669; doi:10.1186/s12934-026-03015-9)
Supplement: Supplementary file 1 — Supplementary Material 1. [file 12934_2026_3015_MOESM1_ESM.docx]

**Supplements to**

**Co-cultivation** **dynamics of the filamentous microorganisms *Aspergillus niger* and *Streptomyces coelicolor* in shake flasks**

Tolue Kheirkhah^1^*, Fangxing Zhang^2^*, Fabia Jaeger^1^, Alexander Gantenbein^1^, Peter Neubauer^1^, Heiko Briesen^2§^, Stefan Junne^1,3§^

*Authors contributed equally

§ Corresponding authors

^1^ Technische Universität Berlin, Institute of Biotechnology, Chair of Bioprocess Engineering,

Ackerstraße 76 ACK24, D-13355 Berlin, Germany

^2^ Technische Universität München, School of Life Sciences Weihenstephan, Chair of Process Systems Engineering, Gregor-Mendel-Straße 4, D-85354 Freising, Germany

^3^ Aalborg University, Department of Chemistry and Bioscience, Niels Bohrs Vej 8, DK-6700 Esbjerg, Denmark

Correspondence to Stefan Junne, Heiko Briesen: [sju@bio.aau.dk](mailto:sju@bio.aau.dk); [heiko.briesen@tum.de](mailto:heiko.briesen@tum.de)

**Table S1** Calculation formula of the morphological feature parameters for classification of *Aspergillus niger* and *Streptomyces coelicolor* in co-culture and the classification accuracy.

|  | Feature parameters | Calculation formula | |
| --- | --- | --- | --- |
|  | Gray value intensity | $I_{avg}=\frac{1}{N}\cdot\sum_{i=1}^{N} \frac{I_{i}(x,y)}{255}$ | |
|  | Equivalent diameter | $d= \sqrt{\frac{4\cdot A_{pellet}}{\pi}}$ | |
|  | Aspect ratio | $AR= \frac{l_{min}}{l_{max}}$ | |
|  | Circularity | $C=\frac{4\cdot\pi\cdot A_{pellet}}{P_{pellet}^{2}}$ | |
|  | Number of pellet cores | $N_{core}$ determined by MATLAB function “bwconncomp” | |
|  | Area proportion of  pellet cores | $\varphi_{core}=\frac{A_{core}}{A_{pellet}}$ | |
|  | Eccentricity of biggest core | ${\Delta s}_{centroid}=\frac{\sqrt{{(\bar{x}_{pellet}-\bar{x}_{max spore})}^{2}+{(\bar{y}_{pellet}-\bar{y}_{max spore})}^{2}}}{d_{pellet}}$ | |
| 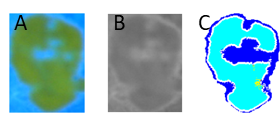 | | A: | raw image of the pellet |
|  |  | B: | grayscale image of the pellet |
|  |  | C: | processed image of the pellet, the dark blue marks the outer contour of the pellet, and the cyan marks the pellet cores |
|  | $I(x,y)$ |  | gray value intensity of pixel at $(x, y)$ |
|  | $l_{min}$ |  | minimal pellet length |
|  | $l_{max}$ |  | maximal pellet length |
|  | $A$ |  | surface area |
|  | $P$ |  | perimeter of pellet |
|  | $(\bar{x}, \bar{y})$ |  | centroid coordination (µm) |

**
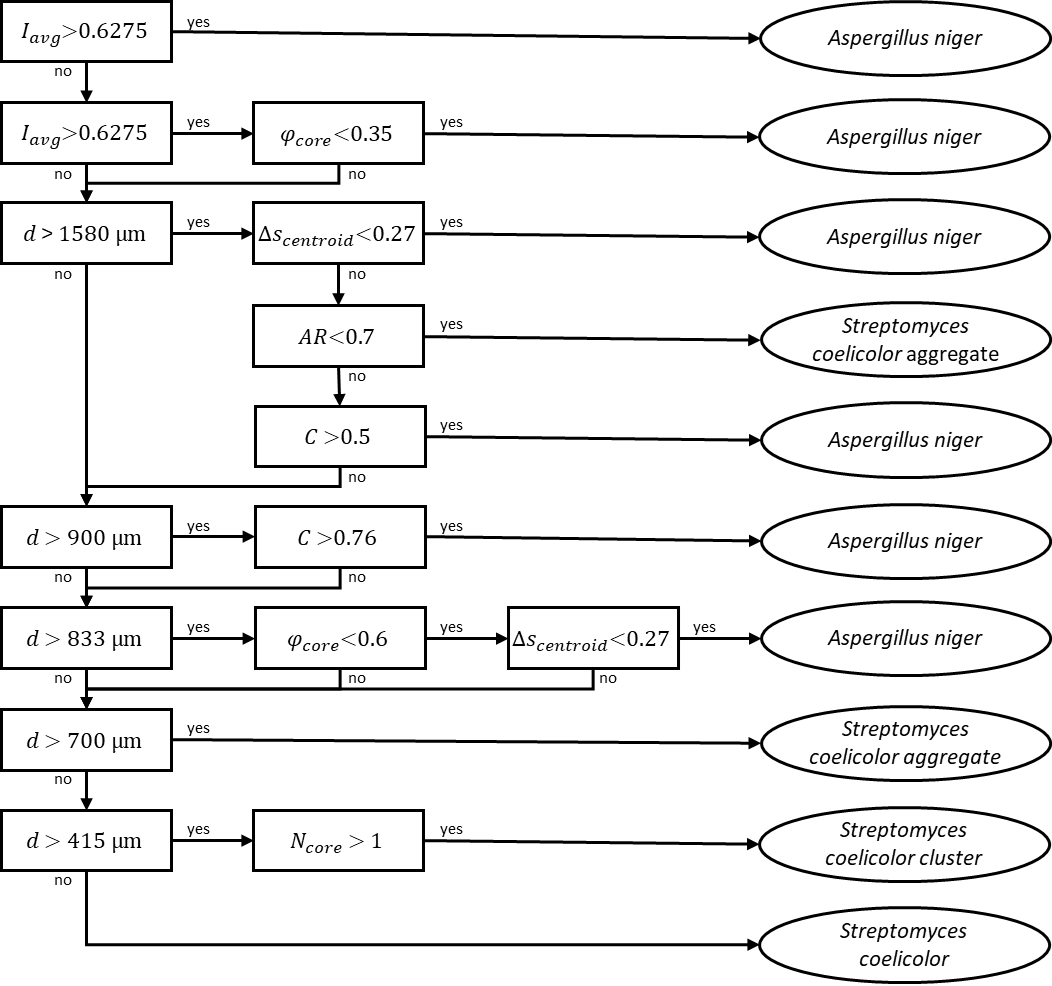
**

**Fig. S1** Morphology criteria for classification of Aspergillus niger and Streptomyces coelicolor pellets. $I_{avg}$ is the average gray value intensity of the pellet, $d\text{ }$the equivalent diameter**,** $AR$ the aspect ratio, $C$ the circularity, $N_{core}$ the number of pellet cores, $\varphi_{core}$ the area proportion of pellet cores, ${\Delta s}_{centroid}$ the eccentricity of the biggest core.

**Table S2** Correlation matrix of the morphological feature parameters.

|  | Gray value  intensity | Equivalent  diameter | Aspect  ratio | Circularity | Number of  pellet cores | Area proportion of pellet cores | Eccentricity |
| --- | --- | --- | --- | --- | --- | --- | --- |
| Gray value  intensity | 1.00 | 0.25 | 0.02 | -0.19 | 0.46 | -0.17 | 0.32 |
| Equivalent  diameter | 0.25 | 1.00 | 0.02 | 0.05 | 0.60 | -0.26 | 0.13 |
| Aspect  ratio | 0.02 | 0.02 | 1.00 | 0.44 | -0.02 | 0.04 | -0.23 |
| Circularity | -0.19 | 0.05 | 0.44 | 1.00 | -0.11 | 0.08 | -0.21 |
| Number of  pellet cores | 0.46 | 0.60 | -0.02 | -0.11 | 1.00 | -0.35 | 0.35 |
| Area proportion  of pellet cores | -0.17 | -0.26 | 0.04 | 0.08 | -0.35 | 1.00 | -0.47 |
| Eccentricity | 0.32 | 0.13 | -0.23 | -0.21 | 0.35 | -0.47 | 1.00 |


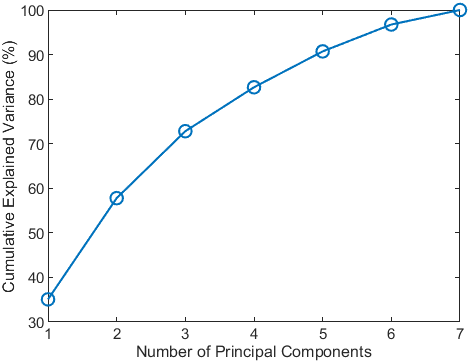


**Fig. S2** Cumulative explained variance by principal components of the morphological feature parameters.

**Table. S3** Loadings of the morphological features on each component.

|  | PC1 | PC2 | PC3 | PC4 | PC5 | PC6 | PC7 |
| --- | --- | --- | --- | --- | --- | --- | --- |
| Gray value intensity | -0.46 | -0.05 | 0.31 | -0.47 | 0.52 | -0.42 | 0.16 |
| Equivalent diameter | 0.34 | -0.43 | 0.41 | -0.38 | 0.17 | 0.49 | -0.35 |
| Aspect ratio | 0.16 | 0.41 | 0.67 | -0.14 | -0.53 | -0.23 | 0.02 |
| Circularity | 0.12 | 0.62 | 0.23 | 0.30 | 0.57 | 0.36 | 0.07 |
| Number of pellet cores | -0.16 | -0.46 | 0.47 | 0.72 | 0.06 | -0.14 | 0.01 |
| Area proportion of pellet cores | 0.56 | -0.21 | 0.01 | -0.05 | 0.10 | -0.10 | 0.78 |
| Eccentricity | -0.54 | -0.05 | 0.11 | -0.09 | -0.28 | 0.61 | 0.48 |

**Table S4** Classification accuracy of *Aspergillus niger* *and Streptomyces coelicolor* pellets using the proposed image analysis pipeline. The values are given in percentages.

|  |  | | | | | Classification accuracy (%) | | | | | | | | |
| --- | --- | --- | --- | --- | --- | --- | --- | --- | --- | --- | --- | --- | --- | --- |
|  | | Shaking velocity [rpm] | Pre-culture volume [mL] | Inoculation ratio [-] (*A. niger* :  *S. coelicolor*) | Flask design | | 0h | 4 h | 12 h | 21 h | 31 h | 46 h | Mean accuracy of experiment |  |
| 1 | | 250 | 50 | 1:1 | BF | | - | 98.8 | - | 98.4 | - | 95.5 | 97.6 |  |
| 2 | | 250 | 50 | 1:1 | BF | | 90.6 | 86.2 | - | 89.3 | 81.8 | 74.4 | 84.5 |  |
| 3 | | 136 | 50 | 1:1 | BF | | - | 95.6 | - | 99.4 | - | 95.4 | 96.8 |  |
| 4 | | 136 | 50 | 1:1 | BF | | 88.0 | 99.4 | 99.2 | 98.2 | 95.8 | 96.4 | 96.2 |  |
| 5 | | 60 | 50 | 1:1 | BF | | - | 95.7 | - | 98.0 | - | 94.1 | 95.9 |  |
| 6 | | 60 | 50 | 1:1 | BF | | - | - | - | - | 100.0 | 100.0 | 100.0 |  |
| 7 | | 60 | 50 | 1:1 | BF | | 96.5 | 94.6 | - | 87.3 | - | 91.1 | 92.4 |  |
| 8 | | 250 | 50 | 1:1 | NB | | - | 87.3 | - | 90.8 | 90.7 | 90.6 | 89.8 |  |
| 9 | | 250 | 50 | 1:1 | NB | | 97.5 | 97.5 | 97.4 | 98.2 | 98.0 | 98.3 | 97.8 |  |
| 10 | | 250 | 50 | 1:2 | NB | | - | 99.7 | - | - | 97.7 | 99.2 | 98.9 |  |
| 11 | | 250 | 50 | 1:2 | NB | | 99.4 | 100.0 | - | 100.0 | 99.6 | 99.6 | 99.7 |  |
| 12 | | 250 | 50 | 1:5 | NB | | 97.8 | 98.2 | - | 98.6 | 95.3 | 95.2 | 97.0 |  |
| 13 | | 250 | 50 | 1:5 | NB | | - | 99.0 | - | - | 92.5 | 92.6 | 94.7 |  |
| 14 | | 250 | 75 | 1:1 | NB | | - | 95.8 | - | 99.3 | - | - | 97.6 |  |
| 15 | | 250 | 75 | 1:1 | NB | | 95.6 | 95.4 | - | 93.3 | 93.1 | 91.3 | 93.8 |  |
| 16 | | 250 | 75 | 1:1 | BF | | 97.2 | 95.3 | 94.0 | 95.7 | - | - | 95.5 |  |
| 17 | | 250 | 75 | 1:1 | BF | | - | 95.3 | - | 92.7 | - | - | 94.0 |  |
|  | |  |  | Mean accuracy of time point | | | 95.3 | 95.9 | 96.9 | 95.7 | 94.5 | 93.8 |  |  |
|  | |  |  |  | | |  |  |  | Overall accuracy | | | 95.1 |  |


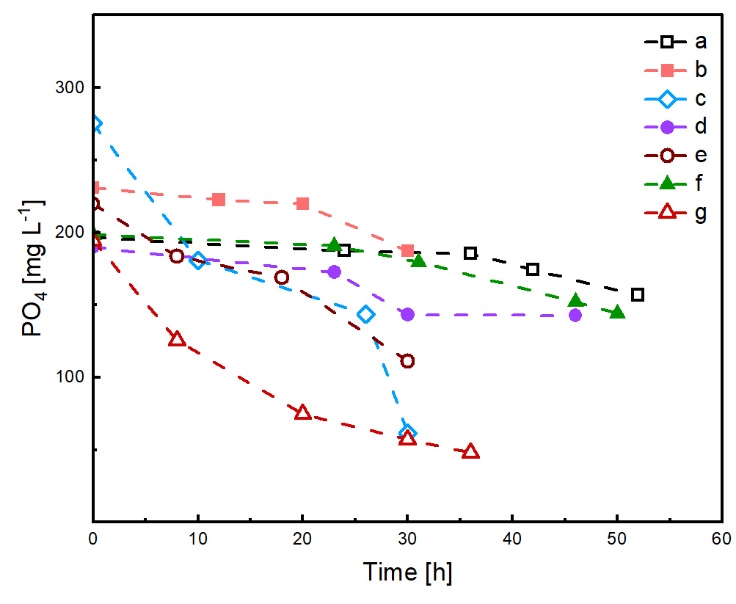


**Fig. S3** Comparison of phosphate concentrations in various inoculation scenarios. a: Axenic culture of Aspergillus niger starting from spores, b: Axenic culture of A. niger from pellets (pre-culture), c: Axenic culture of Streptomyces coelicolor from pellets (pre-culture), d: Co-culture of A. niger spores and S. coelicolor pellets, e: Co-culture of A. niger pellets and S. coelicolor germinated spores, f: Co-culture of A. niger spores and S. coelicolor spores, g: Co-culture of A. niger pellets and S. coelicolor pellets.


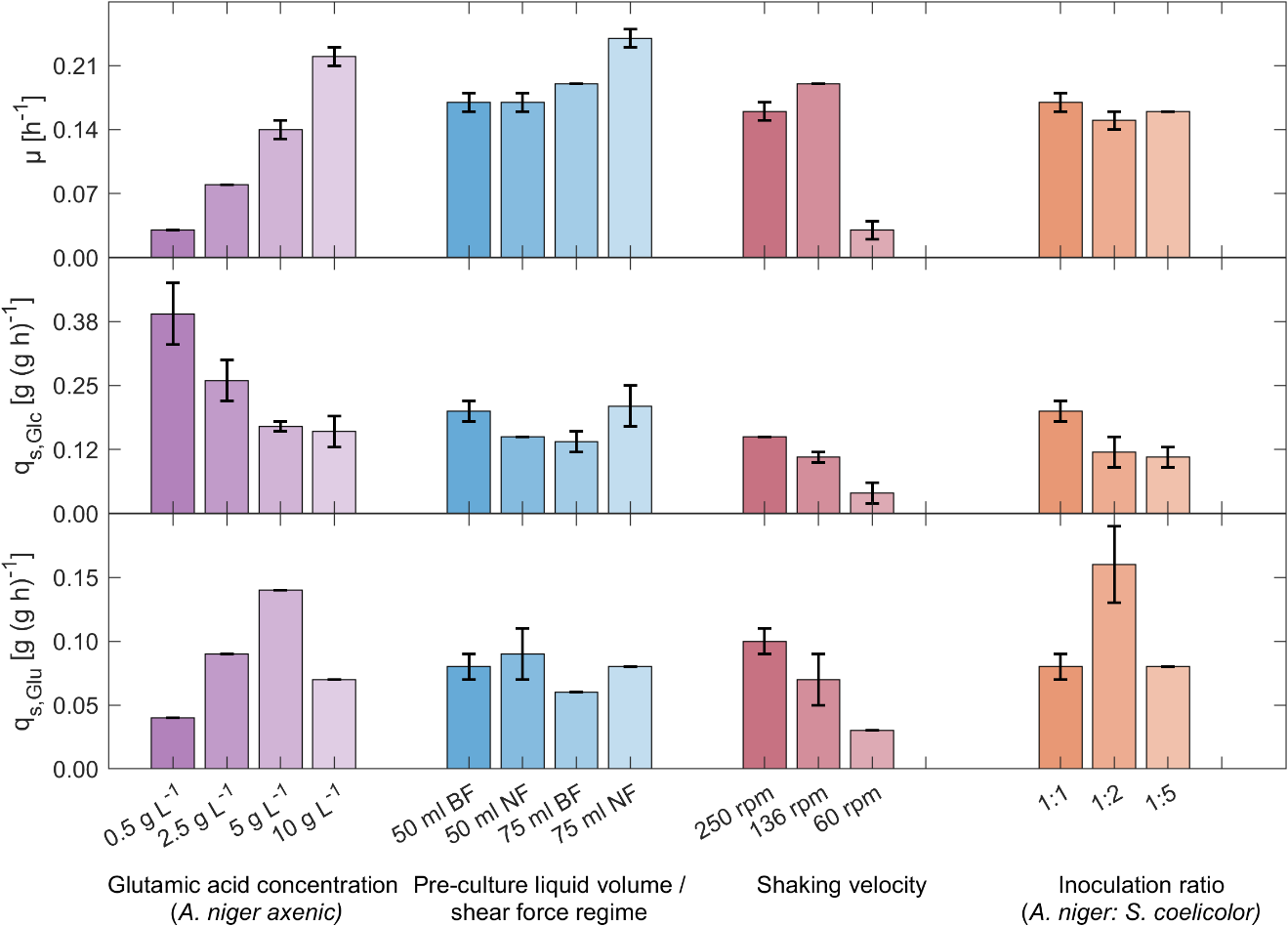


**Fig. S4** Impact of cultivation conditions on process parameters of axenic and co-cultures of *Aspergillus niger* and *Streptomyces coelicolor* in shake flasks. Each experiment was conducted with two biological replicates. Error bars indicate the standard deviation of the replicates.


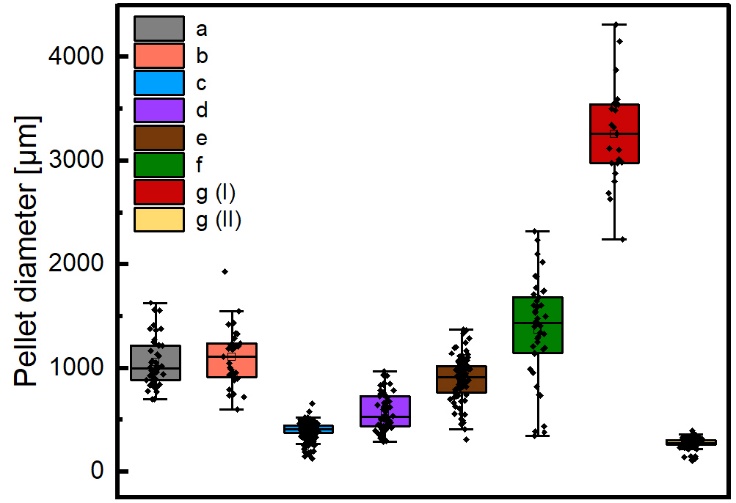


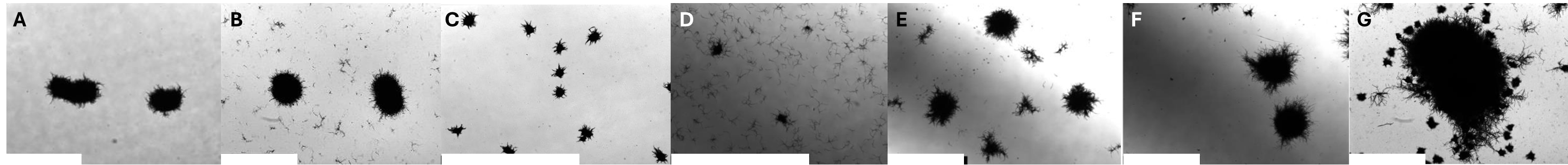


**Fig. S5** Comparison of pellet diameter between axenic and co-cultures at 30 hours cultivation time. a: Axenic culture of Aspergillus niger starting from spores, b: Axenic culture of A. niger from pellets (pre-culture), c: Axenic culture of Streptomyces coelicolor from pellets (pre-culture), d: Co-culture of A. niger spores and S. coelicolor pellets, e: Co-culture of A. niger pellets and S. coelicolor germinated spores, f: Co-culture of A. niger spores and S. coelicolor spores, g: Co-culture of A. niger pellets and S. coelicolor pellets; g (I): A. niger pellets from the co-culture with S. coelicolor pellets, g (II): S. coelicolor pellets from the co-culture with A. niger pellets. The scale bars represent 2.4 mm.


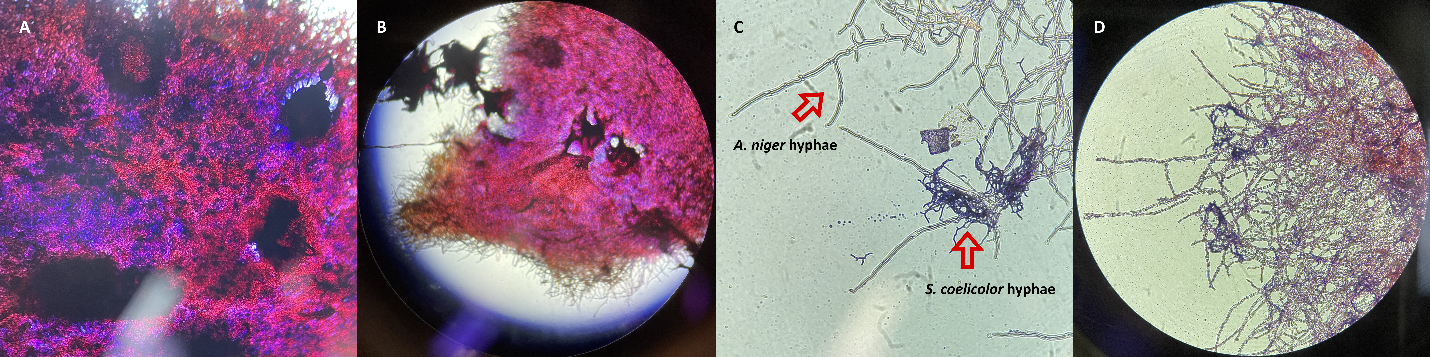


**Fig. S6** A, B: Aggregates of *S. coelicolor* embedded within an *A. niger* pellet, illustrating physical entrapment. C, D: Hyphal growth of *S. coelicolor* at the periphery of an *A. niger* pellet. Images were obtained from various co-cultures after 24 h of cultivation. Samples were Gram-stained and visualized using a manual bright-field microscope. A and B were captured at 40x magnification, and panels C and D at 100x magnification. No exact scale is available, images are used for qualitative description.


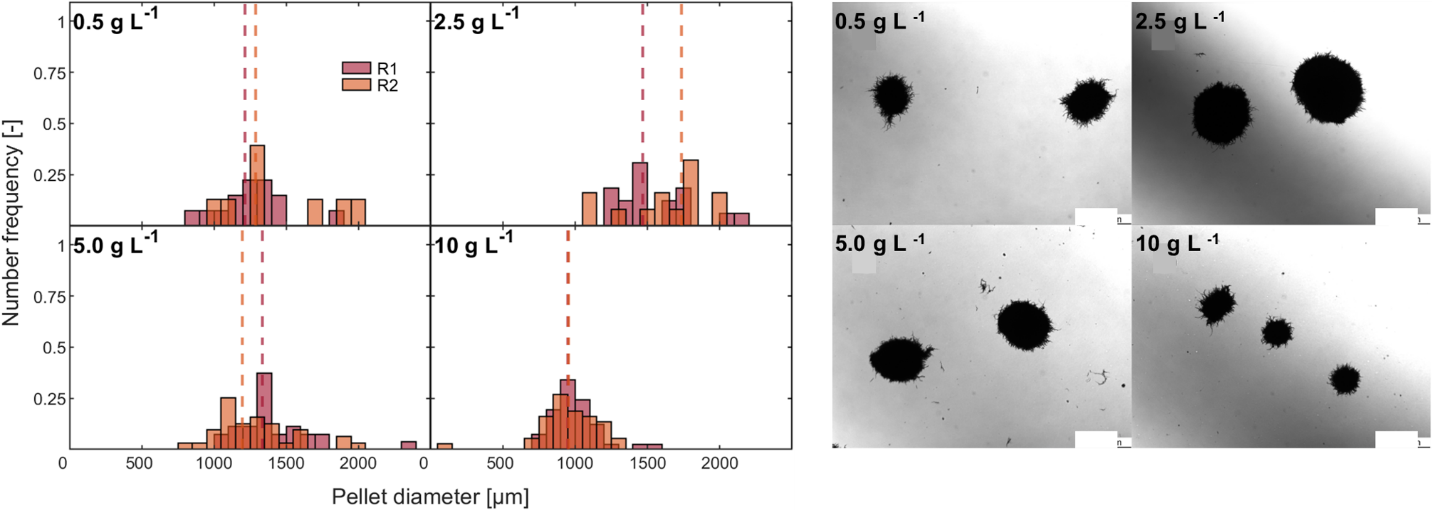


**Fig. S7** Right: Effect of initial glutamic acid concentration (0.5, 2.5, 5.0 and 10 g L^-1^) on the pellet size distribution of Aspergillus niger in shake flask cultivations. Left: Microscopic images of corresponding axenic cultures. Images were taken at 12.5x magnification using a DIC microscope, the scale bar represents 1 mm.

| **Table S5** Statistical measures of pellets heterogeneity of *Aspergillus niger* and *Streptomyces coelicolor* in shake flask under various cultivation conditions. | | | | | | | | | |  |
| --- | --- | --- | --- | --- | --- | --- | --- | --- | --- | --- |
|  |  | | ***Aspergillus niger*** | | | ***Streptomyces coelicolor*** | | | | |
| **Experiment** | **Flask design**  **(BF: baffled. NF: non-baffled)** | | $\boldsymbol{d}_{\boldsymbol{50}}$  **[µm]** | $\boldsymbol{\sigma}_{\boldsymbol{d}}$  **[µm]** | $\boldsymbol{w}_{\boldsymbol{d}\boldsymbol{,}\boldsymbol{norm}}$  **[-]** | $\boldsymbol{d}_{\boldsymbol{50}}$  **[µm]** | $\boldsymbol{\sigma}_{\boldsymbol{d}}$  **[µm]** | $\boldsymbol{w}_{\boldsymbol{d}\boldsymbol{,}\boldsymbol{norm}}$  **[-]** | | |
| ***A. niger* axenic culture: Impact of glutamic acid concentration [g L^-1^]** | | |  |  |  |  |  | |  | |
| **0.5** 46 h | NF | | 1253 | 312.08 | 0.54 | N/A | N/A | N/A | | |
| **2.5** 46 h | NF | | 1601 | 281.84 | 0.44 | N/A | N/A | N/A | | |
| **5.0** 46 h | NF | | 1266 | 269.63 | 0.43 | N/A | N/A | N/A | | |
| **10.0** 46 h | NF | | 952 | 187.19 | 0.38 | N/A | N/A | N/A | | |
| **Pre-culture liquid volume / shear force regime** | | |  |  |  |  |  | |  | |
| **50 mL pre-culture** | BF | |  |  |  |  |  |  | | |
| 4 h |  | | 407 | 194.48 | 1.03 | 208 | 51.19 | 0.61 | | |
| 21 h |  | | 1148 | 218.30 | 0.43 | 191 | 45.51 | 0.59 | | |
| 46 h |  | | 1161 | 429.54 | 0.78 | 192 | 48.02 | 0.56 | | |
| **50 mL pre-culture** | NF | |  |  |  |  |  |  | | |
| 4 h |  | | 465 | 331.43 | 1.89 | 201 | 46.86 | 0.58 | | |
| 21 h |  | | 1461 | 444.29 | 0.72 | 174 | 41.10 | 0.60 | | |
| 46 h |  | | 1023 | 481.00 | 1.38 | 184 | 42.05 | 0.57 | | |
| **75 mL pre-culture** | BF | |  |  |  |  |  |  | | |
| 4 h |  | | 410 | 197.65 | 1.20 | 170 | 37.46 | 0.53 | | |
| 21 h |  | | 754 | 186.17 | 0.63 | 156 | 45.12 | 0.68 | | |
| **75 mL pre-culture** | NF | |  |  |  |  |  |  | | |
| 4 h |  | | 291 | 177.35 | 1.34 | 166 | 30.29 | 0.44 | | |
| 21 h |  | | 782 | 253.13 | 0.79 | 161 | 41.82 | 0.62 | | |
| **Axenic culture and co-culture** | | |  |  |  |  |  | |  | |
| **Axenic culture of *A. niger* from pellets** | NF | |  |  |  |  |  | |  | |
| 4 h |  | | 346 | 213.67 | 1.25 | N/A | N/A | N/A | | |
| 21 h |  | | 443 | 256.42 | 0.83 | N/A | N/A | N/A | | |
| **Axenic culture of *S. coelicoclor* from pellets** | NF | |  |  |  |  |  | |  | |
| 4 h |  | | N/A | N/A | N/A | 176 | 32.15 | 0.43 | | |
| 21 h |  | | N/A | N/A | N/A | 215 | 44.60 | 0.45 | | |
| **Co-culture of *A. niger* pellets to *S. coelicolor* pellets** | NF | |  |  |  |  |  | |  | |
| 4 h |  | | 407 | 194.48 | 1.03 | 208 | 51.19 | 0.61 | | |
| 21 h |  | | 1148 | 218.30 | 0.43 | 191 | 45.51 | 0.59 | | |
| 46 h |  | | 1161 | 429.54 | 0.78 | 192 | 48.02 | 0.56 | | |
|  | | |  |  |  |  |  | |  | |
|  | | |  |  |  |  |  | |  | |
| **Impact of shaking velocity [rpm]** | | |  |  |  |  |  | |  | |
| **250** | BF | |  |  |  |  |  |  | | |
| 4 h |  | | 407 | 194.48 | 1.03 | 208 | 51.19 | 0.61 | | |
| 21 h |  | | 1148 | 218.30 | 0.43 | 191 | 45.51 | 0.59 | | |
| 46 h |  | | 1161 | 429.54 | 0.78 | 192 | 48.02 | 0.56 | | |
| **136** | BF | |  |  |  |  |  |  | | |
| 4 h |  | | 542 | 173.64 | 0.84 | 212 | 44.22 | 0.52 | | |
| 21 h |  | | 1455 | 823.47 | 1.53 | 193 | 39.55 | 0.47 | | |
| 46 h |  | | 834 | 675.04 | 3.83 | 198 | 47.32 | 0.61 | | |
| **60** | BF | |  |  |  |  |  |  | | |
| 4 h |  | | 439 | 388.59 | 2.22 | 217 | 47.68 | 0.57 | | |
| 21 h |  | | 359 | 333.39 | 2.21 | 259 | 60.30 | 0.61 | | |
| 46 h |  | | 948 | 503.95 | 1.73 | 387 | 79.17 | 0.49 | | |
| **Impact of inoculation ratio (*A. niger*: *S. coelicolor*)** | | |  |  |  |  |  | |  | |
| **1:1** | NF | |  |  |  |  |  |  | | |
| 4 h |  | | 465 | 194.48 | 1.03 | 201 | 46.86 | 0.58 | | |
| 21 h |  | | 1321 | 218.30 | 0.43 | 180 | 45.21 | 0.60 | | |
| 46 h |  | | 1023 | 429.54 | 0.78 | 184 | 42.05 | 0.57 | | |
| **1:2** | NF | |  |  |  |  |  |  | | |
| 4 h |  | | 1121 | N/A | N/A | 210 | 43.11 | 0.49 | | |
| 21 h |  | | 2683 | N/A | N/A | 288 | 51.24 | 0.41 | | |
| 46 h |  | | 2288 | 1107.71 | 0.90 | 296 | 59.71 | 0.46 | | |
| **1:5** | NF | |  |  |  |  |  |  | | |
| 4 h |  | | 1138 | N/A | N/A | 218 | 50.97 | 0.52 | | |
| 21 h |  | | 801 | 576.07 | 1.59 | 304 | 64.01 | 0.51 | | |
| 46 h |  | | 708 | 352.16 | 1.08 | 304 | 60.34 | 0.48 | | |
| Median diameter: $d_{50}$ | | Standard deviation: $\sigma_{d}$ | | | Normalized span width: $w_{d,norm}=\frac{d_{90}-d_{10}}{d_{50}}$ | | | | |  |
| N/A: error due to an inefficient number of pellets | |  | | |  | | | | | |

**Table S6** P-values from one-way and two-way ANOVA followed by Tukey honest significant difference test, evaluating the influence of different cultivation parameters under the indicated cultivation time points.

|  |  | tp | $p_{A. niger}$ | $p_{S. coelicolor}$ |
| --- | --- | --- | --- | --- |
| One-way-anova | Glutamic acid |  | 0.0197 | - |
|  | Shaking velocity | 21 | 0.0001 | 0.0334 |
|  | Inoculation ratio | 31 | 0.0231 | 0.0044 |
|  | Mono-/Co-culture | 21 | 0.0073 | 0.5264 |
| Two-way anova | Pre-culture volume | 21 | 0.0017 | 0.0411 |
|  | Baffle | 21 | 0.0749 | 0.4614 |
|  | Pre-culture volume:baffle | 21 | 0.1156 | 0.2588 |

**Table S7** Mathematical formulations of process parameters.

| Process parameter | Formula |
| --- | --- |
| Specific growth rate | $\mu=\frac{1}{X}\frac{dX}{dt}$ |
| Specific substrate consumption rate | $q_{s}=-\frac{1}{X}\frac{dS}{dt}$ |
| Consumption rate | $r_{s}=q_{s}\cdot X$ |
| Yield coefficient | $Y_{X/s}=-\frac{dX}{dS}$ |


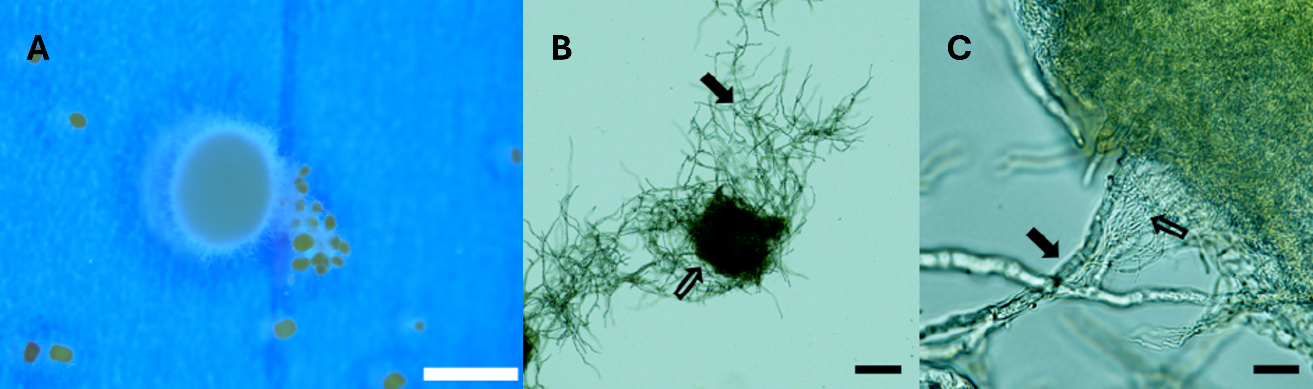


**Fig. S8** A: Microscopic image of Streptomyces coelicolor and Aspergillus niger pellets in co-culture with a baffled flask using 50 mL pre-culture medium volume. The image shows Streptomyces coelicolor pellets intertwined to Aspergillus niger hyphae. It was taken 21 hours after inoculation with a stereo microscope. Scale bar represents 1 mm. B, C: Microscopic images of an S. coelicolor pellet (unfilled arrow) in physical contact with A. niger hyphae (filled arrow). Images were captured using a Nikon Eclipse Ti2 inverted microscope using 100x magnification for B and 1000x for C. Scale bars indicate 100 μm (B) and 10 μm (C).


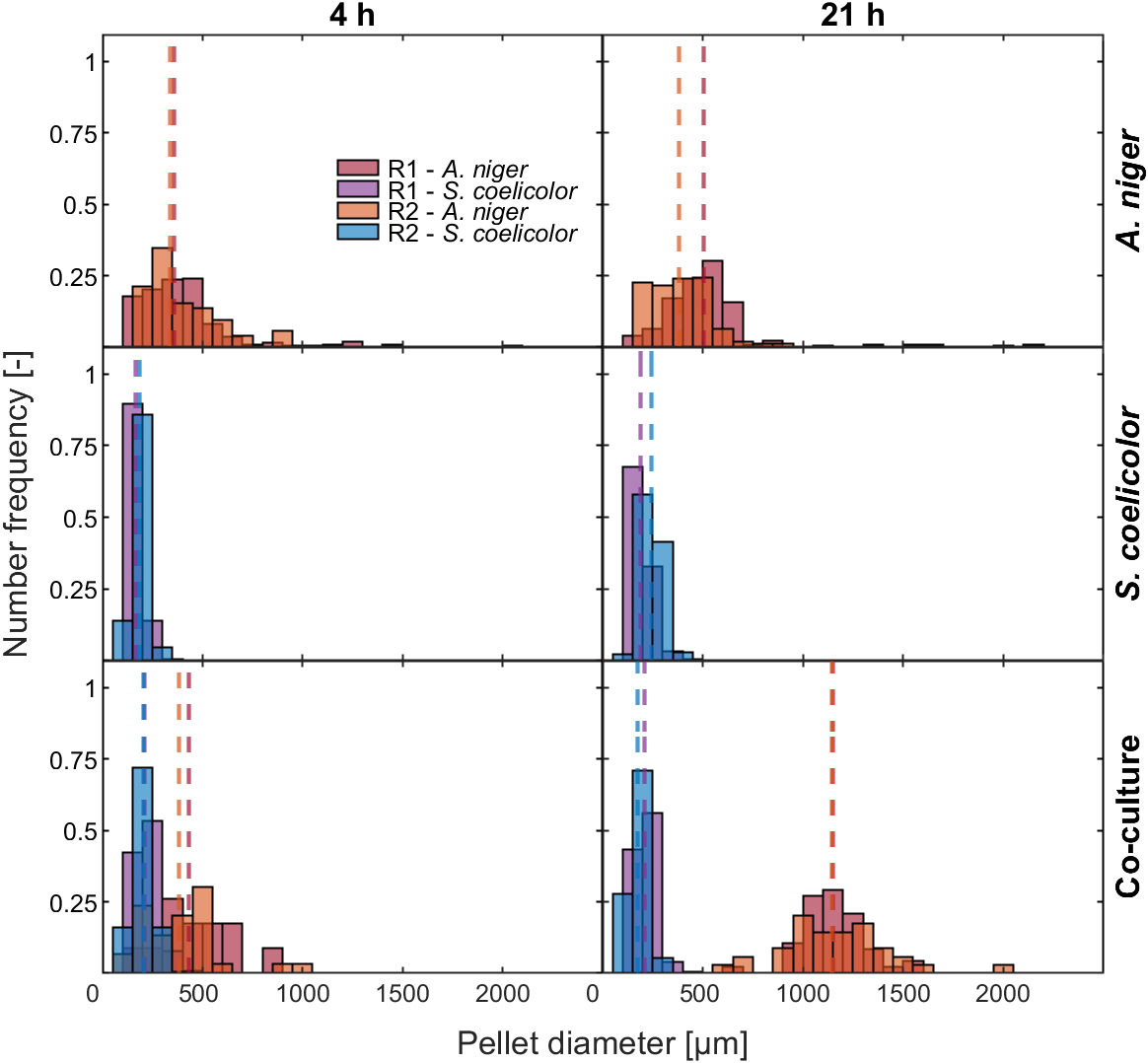


**Fig. S9** Time course development of pellet size distribution of Aspergillus niger and Streptomyces coelicolor, comparison of axenic cultures with the co-culture. Data is presented as histogram plots (number frequency q0, bars) and D50 (median pellet diameter, lines). R1 and R2 are biological replicates.


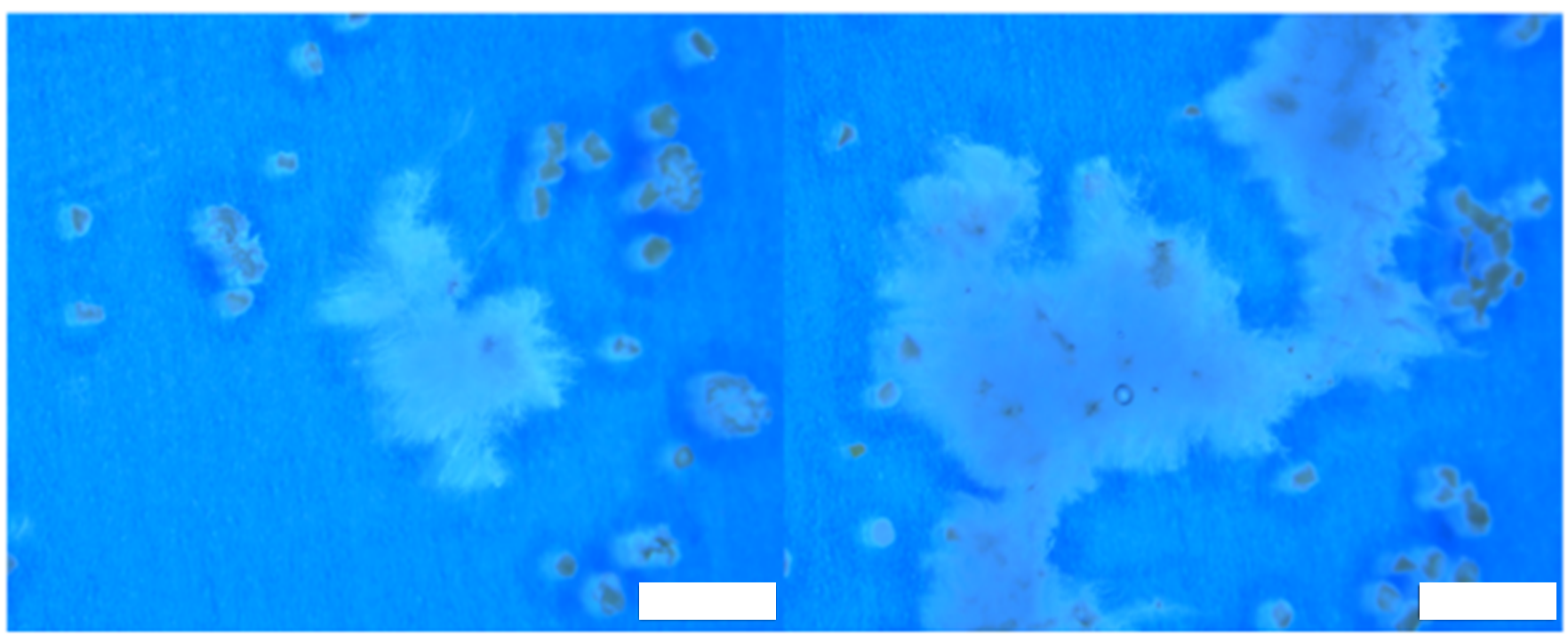


**Fig. S10** Microscopic images of Aspergillus niger (large clump) and Streptomyces coelicolor pellets in the co-culture at 60 rpm. Images were taken 46 hours after inoculation using a stereo microscope. The scale bar represents 2 mm.
